# Recent Advancements in Gel-Based Flexible Electronic Sensors

**DOI:** 10.3390/gels12050402

**Published:** 2026-05-06

**Authors:** Vineet Kumar, Sang-Shin Park

**Affiliations:** School of Mechanical Engineering, Yeungnam University, 280 Daehak-Ro, Gyeongsan 38541, Gyeongbuk, Republic of Korea; vineetfri@gmail.com

**Keywords:** gels, soft electronics, electronic skin, bioprinting, portable sensors

## Abstract

Gel-based flexible electronic sensors have emerged as a transformative class of materials for next-generation applications. These applications are wearable electronics, soft robotics, electronic skin (e-skin), and healthcare monitoring systems. Owing to their intrinsic softness, stretchability, and biocompatibility, gels provide an ideal platform for constructing highly deformable and skin-conformable sensing devices. This paper provides insight into emerging fabrication techniques, including 3D printing, bioprinting, and microfabrication. These techniques have facilitated the creation of complex architectures with improved sensitivity and scalability. The review also focuses on recent advancements that have focused on overcoming traditional limitations. These limitations are poor mechanical strength, dehydration, limited environmental stability, and low sensitivity. In particular, the incorporation of conductive fillers and ionic species has enabled a range of sensing mechanisms. These mechanisms include piezoresistive, capacitive, piezoelectric, and ionotronic responses. Therefore, it allows for the accurate detection of strain, pressure, temperature, and biochemical signals. Finally, this review provides a summary of future research, which is expected to focus on multifunctional integration, sustainable materials, and intelligent data processing. It provides pathways to the widespread adoption of gel-based flexible electronic sensors in both consumer and clinical applications.

## 1. Introduction

Gel-based flexible electronic sensors are soft devices that utilize gel materials as the active or functional layer to detect external stimuli [1]. Gels are particularly suitable for flexible electronics due to their softness, stretchability, high water content, and excellent biomechanical compatibility. Based on the dispersion medium, gels are commonly classified into hydrogels, which use water as the solvent [2], and organogels, in which organic liquids are trapped within the polymer network. Hydrogels are especially important in biomedical and soft electronic applications because of their biocompatibility, high water content, and flexibility [3]. In these sensors, gels form a three-dimensional polymer network capable of retaining large amounts of liquid while maintaining structural integrity. This structure enables efficient ion or electron transport, which allows gels to convert external stimuli such as pressure, strain, bending, temperature, or humidity into measurable electrical signals [4]. Gel-based sensors typically operate through changes in electrical resistance or ionic conductivity. The main mechanism includes piezoresistive/piezoelectric responses when the gel network is deformed. When mechanical stress is applied, the internal polymer network and conductive pathways change, resulting in variations in electrical properties [5]. One major advantage of gel-based sensors is their high flexibility and stretchability. This allows them to maintain stable performance under large deformation.

Gels exhibit self-healing ability, transparency, biocompatibility, and high sensitivity. Owing to these properties, gel-based flexible sensors have been widely applied in health monitoring, human motion detection, electronic skin (e-skin), soft robotics, pressure sensing, and environmental sensing [6]. They are also being explored for use in battery-free, wireless sensing systems, where soft materials can conform to irregular surfaces and continuously monitor physiological signals. Gels are typically formed through the physical or chemical crosslinking of polymer chains [7]. In physical gels, the network is formed through weak or ionic interactions, including hydrogen bonding and van der Waals forces. In contrast, chemical gels contain strong covalent crosslinks that connect the polymer chains, resulting in a more permanent and stable structure [8]. Moreover, gels possess several unique properties, such as high swelling capacity, viscoelastic behavior, permeability, and responsiveness to external stimuli. These characteristics make them suitable for a wide range of applications, including portable sensors [9,10]. Their structure provides both mechanical flexibility and electrical functionality. The soft polymer network allows the material to undergo large deformations—such as bending, stretching, and compression—without losing functionality [11]. Another important property is high sensitivity to mechanical stimuli [12].

Gel-based electronic sensors exhibit excellent biocompatibility and softness, making them ideal for wearable health monitoring and biomedical applications. Their mechanical properties are often similar to those of biological tissues, enabling comfortable long-term contact with the body [13]. In addition, many gels demonstrate self-healing capability, whereby damaged networks can recover their structure through reversible physical interactions. This property enhances the durability and lifespan of flexible sensors. Gels also provide high ionic or electronic conductivity, depending on the incorporated conductive fillers or ions [14]. The presence of mobile ions or conductive nanomaterials enables efficient signal transmission and improves sensor performance. Other notable properties include transparency, high water retention, environmental responsiveness (to temperature, pH, or humidity), and tunable mechanical strength. These characteristics allow gel-based sensors to be engineered for specific sensing applications [15].

Recent progress in gel-based flexible electronic sensors is characterized by several key innovations that distinguish modern systems from conventional soft sensors. These advances span material design, sensing mechanisms, environmental adaptability, and system integration. For example, the use of dynamic and reversible crosslinking enables self-healing and enhances the toughness of composites. However, to the best of the author’s knowledge, the use of gel-based materials in electronic sensors is not yet fully understood. Therefore, this work presents a novel methodology for fabricating gel-based materials using next-generation techniques, including 3D printing, bioprinting, and microfabrication. This study is followed by an analysis of the mechanical, electromechanical, and thermal behavior of these gel-based composites. Finally, their applications—such as wearable electronics, soft robotics, electronic skin, and healthcare monitoring systems—are discussed. A summary of the work is provided in Table 1.

The first part of this paper reports the fabrication process of the gel-based sensors. This involves the development of these sensors through traditional pathways like molding or solution casting. This section is followed by studying advanced fabrication processes. These are 3D printing and bioprinting, which produce gel-based sensors with high precision and robust performance. The second part of this paper illustrates the key properties of these sensors. These properties are mechanical performance, electromechanical properties, electrical properties, and finally, thermal properties. This section further describes how changes in these properties affect the sensor’s performance. These properties also show how the addition of filler can influence the sensor’s mechanical strength and piezo-resistivity. The final part of the review reports the use of these gel-based sensors for a particular application of interest. These applications are wearable sensing, healthcare technology, and human–machine interface-related applications. The study of self-powered sensors includes details about their electrical output in the form of output voltage and power density. Similarly, the healthcare studies include heartbeat monitoring, mental health, fitness trackers, and tissue engineering. Moreover, the human–machine interface prospects are reported and studied in detail. They involve studies on electronic skin, soft robotics, tactile sensing, and finally, photo-actuators. Finally, this review paper adds a discussion on the merits and challenges of these gel-based sensors for industrial use. Some of the challenges include the poor filler dispersion, weaker interfaces, and low compatibility among fillers with the gel matrix.

## 2. Fabrication Process

### 2.1. Traditional Fabrication

The fabrication of gel-based electronic sensors generally involves the preparation of a polymer gel network, followed by the incorporation of conductive components and, finally, the integration of electrodes to create a flexible sensing device. The process is designed to produce soft materials with stable mechanical and electrical properties suitable for sensing applications [16]. The first step typically involves preparing the polymer precursor solution. In this stage, polymer materials such as polyvinyl alcohol (PVA), polyacrylamide (PAAm), or other gel-forming polymers are dissolved in water or organic solvents. Crosslinking agents, initiators, and additives may also be introduced to control the formation of the gel network. Next, conductive materials or ions are incorporated into the precursor solution to provide electrical conductivity [17]. These may include conductive fillers such as graphene, carbon nanotubes, metal nanoparticles, MXene, or ionic salts. The mixture is usually stirred or ultrasonicated to ensure uniform dispersion of the conductive components within the polymer matrix. The third step is gel formation through crosslinking. This can occur via chemical polymerization or physical crosslinking methods such as freeze–thaw cycles, ionic interactions, or hydrogen bonding. During this process, a three-dimensional polymer network forms, trapping the solvent and conductive components within the structure [18]. After gel formation, the material is typically molded or cast into desired shapes or thin films, depending on the sensor design. Thin layers are often preferred for flexible electronic sensors because they enhance sensitivity and mechanical flexibility. Finally, flexible electrodes made from materials such as silver nanowires, carbon films, or metal foils are attached to the gel layer [19]. The assembled structure may also be encapsulated with protective layers to improve durability and prevent dehydration.

There are various mechanisms to fabricate gel-based sensors through traditional methods. They are solution casting, molding, dip coating, and layer-by-layer assembly. The solution casting and molding involve the addition of polymer precursors, crosslinkers, and fillers, which are dispersed in a solvent to form a homogeneous solution [20]. This solution is poured into a mold and kept in the air to remove the solvents. The final crosslinked sample is taken out of the molds and studied for performance on different measures. For the mold-based polymerization and crosslinking mechanism, the monomers were injected into the molds, followed by in situ polymerization and crosslinking. This crosslinking can occur through chemical or physical crosslinking. The chemical crosslinking initiates chemical reactions that result in the formation of permanent networks with good stability [21]. In addition to this, the physical crosslinking is driven by non-covalent interactions, enabling reversible and self-healing properties. These non-covalent interactions are hydrogen bonding, ionic bonding, and hydrophobic associations. Here, the molds define the geometries while crosslinking mechanisms govern property tuning. These properties are elasticity, strength, and durability. In the dip-coating mechanism, a substrate is immersed in a gel precursor solution. Then, a thin liquid film adheres to the surface due to viscosity and capillary forces. This film was kept in the air for drying and crosslinking, resulting in a uniform gel layer. The thickness of this layer can be controlled by repeating the dipping and final testing for performance on different measures [22]. Finally, the layer-by-layer assembly involves the deposition step to form a thin layer following different interactions. These interactions are electrostatic attractions, hydrogen bonding, and other intermolecular forces. After repeating the process many times, the resulting multilayer gel film was obtained with nano-scale control over composite thickness.

There are various merits of fabricating the gel-based sensors through traditional technology. They are simple and accessible, cost-effective, and have material versatility and uniform bulk structures [23]. In detail, the traditional fabrication involves solution casting, molding, and curing, which are simple and do not require costly equipment. Therefore, their cost-effectiveness makes them attractive for large-scale production of gel-based sensors. The traditional fabrication of gel-based sensors also allows for a cheap way to control loadings into gel matrices to tailor sensing performance [24]. Similarly, the traditional method supports the fabrication of gel sensors through uniform bulk structures. This will assist in achieving uniform properties and stable baseline sensor performance. Despite having so many merits, the traditional fabrication method also has some shortcomings. These are poor structural resolution, poor spatial heterogeneity, the filler dispersion challenge, and integration challenges. The limited resolution restricts the ability to fabricate complex structures useful for tuning the homogeneity at the micro- and nanoscale. This makes it difficult to create the desired tuned patterns required for multi-model and signal decoupling. Reproducing the structure of these sensors is tough, as the designs are sensitive to humidity and manual processing steps. This process significantly affects the gel-based sensor performance and thus is a big challenge for traditional fabrication [25]. The integration is also a challenge that persists when combining gels with rigid components during assembly. These include electrodes, circuits, or encapsulation layers that lead to mechanical mismatch and reduced device durability. Finally, the processing time in traditional fabrication is high, especially for the multi-layered or chemically crosslinked systems that require a long curing time.

### 2.2. Fabrication Through 3D Printing

The 3D printing of gels is an advanced fabrication technique used to create complex, customizable, and highly precise gel structures for various applications, including flexible electronics, biomedical devices, soft robotics, and tissue engineering. This method enables the layer-by-layer deposition of gel precursors or gel inks to form three-dimensional structures with controlled geometry and material properties. The fabrication process typically begins with the preparation of printable gel inks [26]. These inks are formulated by mixing polymers such as polyacrylamide, polyethylene glycol, alginate, or other gel-forming materials, along with solvents, crosslinking agents, and, in some cases, conductive fillers such as graphene, carbon nanotubes, or metallic nanoparticles. The ink must possess appropriate viscosity and rheological properties to ensure smooth extrusion and structural stability during printing [27]. In the next step, the gel ink is loaded into a 3D printer, commonly using extrusion-based techniques such as direct ink writing (DIW) or fused deposition-based systems. During printing, the gel ink is deposited layer by layer according to a computer-aided design (CAD) model, allowing precise control over the shape, size, and internal architecture of the printed structure. After deposition, the printed gel structure undergoes crosslinking or curing to stabilize the polymer network. Crosslinking can occur through various mechanisms, including chemical reactions, UV curing, ionic crosslinking, or thermal treatment. This step solidifies the gel network and ensures the mechanical stability of the printed object [28]. In some cases, post-processing treatments are performed to improve the mechanical strength, electrical conductivity, or overall functionality of the printed gels. These treatments may include additional curing, solvent exchange, freeze–thaw cycles, or the incorporation of conductive materials.

**Figure 1:**
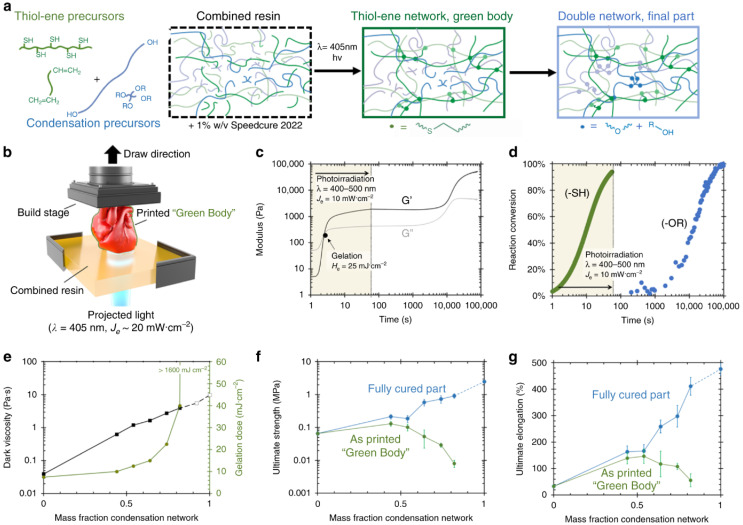
(**a**) illustration of photocured 3D printing resins. After exposure to light, the thiol-ene network crosslinks trap condensation components, thereby crosslinking at room temperature to form secondary networks; (**b**,**c**) an overview of the SLA printing process and rheological behaviors, respectively; (**d**) development of two networks during the printing process; (**e**) the dark viscosity and gelatin dose of resins used against mass fractions of two networks; and (**f**,**g**) tensile strength and elongation at break of the two networks. Reproduced from [36].

Figure 1 illustrates that the rapid advancement of additive manufacturing (3D printing) has enabled robust fabrication processes, simplifying the creation of complex and customized structures for applications such as soft robotics, wearable electronics, biomedical devices, and flexible sensors. Among various material systems, silicone elastomers are widely favored due to their excellent flexibility, biocompatibility, chemical stability, and thermal resistance. However, conventional silicone materials often suffer from low mechanical strength and limited toughness [29], which restricts their use in load-bearing and highly deformable applications. To address these limitations, the concept of double-network (DN) materials has been introduced into silicone systems. Double networks typically consist of two interpenetrating polymer networks with contrasting properties: a rigid, highly crosslinked first network that provides structural integrity, and a soft, stretchable second network that dissipates energy under deformation. This synergistic combination results in materials with exceptional toughness and high durability [30]. However, integrating double-network design with 3D printability remains a significant challenge, as it requires careful control over rheological behavior, curing kinetics, and network formation. Recent developments have enabled the formulation of printable silicone inks that can be extruded with high fidelity and subsequently form robust double-network structures either during or after printing. Strategies such as dual-curing mechanisms (e.g., thermal and UV curing), dynamic crosslinking, and the incorporation of reinforcing fillers have been employed to achieve this balance. These advancements make such materials ideal for fabricating customized soft devices that offer both mechanical robustness and geometric precision [31]. Target applications include soft actuators, stretchable electronics, biomedical implants, and protective components in flexible systems.

Various mechanisms of 3D printing are used for fabricating gel-based composites. These are direct ink writing, the photo-polymerization mechanism, and the formation of conducting networks during printing. In direct ink writing, the gel-based inks are extruded through nozzles under applied pressure. Here, the main mechanism involves the shear-thinning behavior, in which the ink properties, such as viscosity, decrease under shear stress [32]. Once the deposition process is finished, the gel-based material recovers viscosity rapidly to maintain the printed shapes. These mechanisms depend on viscosity, recovery, and the balance between viscosity and shape retention. In another photo-polymerization mechanism, the light-induced polymerization of light-sensitive gel precursors was evidenced. The light-dependent reactive species trigger the crosslinking of monomers. This mechanism depends on light absorption, the generation of reactive radicals, and chain propagation [33]. Finally, the incorporation of conductive fillers like MWCNT or graphene leads to enhanced properties. During printing mechanisms, the shear forces can align fillers, thereby creating anisotropic conductivity. Finally, the post-processing mechanisms involve improved crosslink density, removal of residual solvent, and improvement of mechanical strength.

There are various merits of using 3D printing technology for fabricating gel-based sensors. These are structural complexity control, customization, efficient prototyping, and the tunable properties of the gel sensors. Structural complexity control can be achieved by producing intricate geometries, porous networks, and tunable structures. These features cannot be achieved through traditional methods like molding or solution casting. This precise tuning through 3D printing can support the development of advanced functionalities like electronic skin or multimodal sensing systems. Three-dimensional printing also enables the creation of microstructural features that produce high sensitivity, signal stability, and controlled crack formations. Three-dimensional printing also offers efficient prototyping, as material is deposited as needed [34]. This, finally, enhances sustainability and accelerates the development cycles. Despite having so many merits, 3D printing has various limitations, such as printing speed, material compatibility, post-processing requirements, and cost. Here, the printing speed and scalability are critical, especially for high-resolution or large-area devices. In addition, 3D printing processing time is long, thereby hindering industrial adoption. Another challenge is that post-processing requirements, like curing, crosslinking, and solvent exchange, are difficult. These are critical and have resulted in the introduction of defects, shrinkage, or mechanical instability under mechanical deformations. Finally, the use of expensive equipment and technical expertise requirements for using this technique are critical.

Quantitative benchmarking provides a data-driven framework to evaluate the stereolithography (SLA) and direct ink writing (DIW) printing tools. Both of them provide robust merits and trade-offs when used in gel-based sensors. For example, in terms of resolution, SLA is able to achieve up to 50 µm resolution, while DIW can achieve from ~50 to 300 µm. However, these properties depend on the nozzle size and ink rheology of the machine used in printing. In terms of mechanical performance, DIW-printed gels often retain higher stretchability (>200–500%). In addition to this, SLA gels may exhibit higher stiffness but lower elongation due to dense photopolymer networks. In terms of sensing performance, DIW allows percolated networks, thereby yielding better gauge factors compared to SLA systems. Finally, the SLA-printed gel sensors can exhibit high precision, while DIW sensors are more commonly adopted for large-area fabrications [35]. Overall, DIW and SLA transform fabrications into engineered choices as needed for specific applications.

### 2.3. Bioprinting

Bioprinting for gels is a transformative technology that combines material science, biology, and additive manufacturing. It enables the fabrication of soft, functional systems with applications ranging from healthcare to flexible electronics, making it a key tool in next-generation smart materials and devices. By integrating material design, printing strategies, and post-processing, bioprinting facilitates the development of advanced, flexible, and multifunctional sensing platforms for wearable, biomedical, and soft robotic applications [37]. Bioprinting involves the layer-by-layer deposition of bio-inks, in which gels (hydrogels) serve as the primary matrix. These bio-inks contain polymer networks, conductive fillers (such as graphene or carbon nanotubes), and biological components, including cells and biomolecules. Gels are widely used in bioprinting due to their high water content, flexibility, softness, and biocompatibility. Moreover, they exhibit tunable properties, such as optimized mechanical strength and suitable electrical conductivity [38]. Common bioprinting techniques include inkjet printing, laser-assisted printing, and light-based printing. These methods are frequently employed in tissue engineering, wearable sensors, electronic skin, and drug delivery systems.

**Figure 2:**
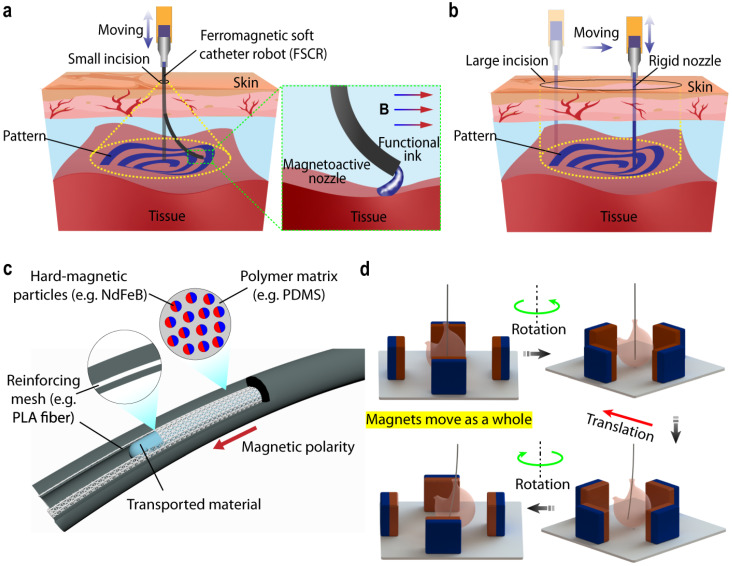
(**a**) Schematic description of minimally invasive bioprinting with functional inks inside the human body; (**b**) schematic illustration of traditional printing systems using a rigid nozzle with large incision; (**c**) illustration of ink composited of soft polymer matrix, dispersed hard magnetic particles and PLA as reinforcing mesh; and (**d**) control of ink by digital data through rotation and translation of permanent magnets. Reproduced from [48].

Figure 2 illustrates that minimally invasive bioprinting has emerged as a transformative approach in biomedical engineering, enabling the in-situ fabrication of biological structures directly within the human body. This technique aims to overcome the limitations of conventional bioprinting, such as the need for open surgical access and the difficulty of printing within confined or delicate anatomical environments. However, a key challenge lies in developing highly controllable, flexible, and biocompatible delivery systems [39] capable of navigating complex internal pathways while maintaining precise printing functionality. In this context, ferromagnetic soft catheter robots have gained significant attention as a promising solution. These systems combine soft robotic architectures with ferromagnetic materials, enabling remote, wireless actuation and navigation using externally applied magnetic fields [40]. Unlike traditional rigid catheters, soft catheter robots can safely adapt to curved and sensitive biological environments, reducing the risk of tissue damage during operation. The integration of ferromagnetic particles within elastomeric or hydrogel-based matrices is critical, as it enables the catheter to respond dynamically to magnetic fields through controlled bending, twisting, and locomotion. By modulating the field strength and gradients, precise positioning and steering of the catheter tip can be achieved, which is essential for accurate bioprinting [41]. This level of control allows the system to deliver bio-inks, cells, or therapeutic agents directly to targeted sites.

Furthermore, these robotic systems can be equipped with micro-extrusion or inkjet-based bioprinting modules, enabling localized deposition of biomaterials for tissue repair, regeneration, or drug delivery. The soft and compliant nature of the catheter ensures conformal contact with tissue surfaces. The use of ferromagnetic soft catheter robots offers several advantages, including wireless operation, high maneuverability, reduced invasiveness, and real-time controllability [42]. These features make them particularly suitable for applications in vascular, gastrointestinal, and other minimally accessible regions. The main mechanism of the fabrication is through the layer-by-layer deposition of bio-inks. These bio-inks are composed of hydrogels, fillers, and living cells. These bio-inks are shear-thinning and viscoelastic materials that flow under stress and rapidly recover to their original position once the deposition is completed [43]. The presence of electrically conducting fillers in bio-inks helps them to enable sensing. This printing process involves tools like inkjet bioprinting or laser-assisted printing. The inkjet printing ejects droplets in a controlled state that assists high-resolution patterning. An important aspect of these mechanisms is crosslinking, which stabilizes the overall printing structures. This crosslinking can be physical or chemical, depending upon the desired functionality and nature of curing agents used during fabrication. Rapid crosslinking ensures shape fidelity, mechanical robustness, and the long-term stability of the sensor [44]. This is particularly good for wearable applications where localized sensitivity is important.

There are various merits to using bioprinting for the fabrication of gel-based sensors for robust performance. These are biocompatibility, bio-integration, precise spatial control, multi-component integrations, and personalized healthcare. Therefore, bioprinting enables the fabrication of heterogeneous and multilayered structures. These structures help to design sensors with multifunctionality [45]. These are also capable of detecting various signals simultaneously. Bioprinting also supports precise spatial control, which enables the development of robust performance as sensors. This technique also enables personalized devices as sensors. These devices allow for the capture of individual physiological data, thereby improving control and accuracy in wearable devices. The use of hydrogel-based bio-inks presents an opportunity to obtain excellent mechanical compliance. This ensures good contact with skin or tissues while minimizing damage to the surrounding tissues. Despite so many merits, bioprinting also suffers from some limitations [46]. These are bio-ink formation constraints, low mechanical strength, poor scalability and regularity, and ethical considerations. Medical-grade materials must be used in bio-ink formation, and these make this method expensive. Moreover, the materials used in bioprinting must satisfy cell viability and printing requirements. Most of the bioprinted hydrogels have low mechanical strength, which involves significant mechanical stress or quick fracture even under small deformations. Moreover, their scalability and reproducibility remain challenging. This is because it is critical to maintain consistent cell distribution and structural integrity [47]. Finally, the medical regulatory requirements associated with living cell protocols must be satisfied.

## 3. Properties

### 3.1. Mechanical Properties

Mechanical properties are critical to the performance of gel-based sensors in flexible and wearable applications. These sensors are designed to mimic soft biological tissues, offering a combination of elasticity, flexibility, and durability. One of the most important properties is high stretchability, which allows gels to undergo large strains (often several hundred percent) without mechanical failure. This behavior arises from the three-dimensional polymer network and the presence of a liquid phase, which enables chain mobility and deformation under stress. Gel-based sensors also exhibit a low Young’s modulus, making them soft and compliant [49]. This property allows them to conform closely to irregular surfaces, such as human skin, thereby improving signal accuracy and user comfort in wearable devices. Another key feature is excellent elasticity and recovery behavior. After deformation (stretching, bending, or compression), gels can return to their original shape with minimal permanent deformation, ensuring repeatable sensing performance under cyclic loading conditions. These materials exhibit viscoelastic behavior, meaning they respond both elastically (solid-like) and viscously (fluid-like). This property influences time-dependent deformation, hysteresis, and energy dissipation, which are important factors in dynamic sensing applications [50]. Gel-based sensors also demonstrate good fatigue resistance, enabling them to withstand repeated mechanical loading over long periods without significant performance degradation. This is essential for applications involving continuous motion, such as human activity monitoring. Additionally, many gel systems possess self-healing capability, in which the polymer network recovers after mechanical damage through reversible interactions [51]. The tensile strength and toughness of gels can be tuned by adjusting the polymer composition, crosslinking density, and the incorporation of reinforcing fillers such as graphene or carbon nanotubes. Advanced designs, such as double-network or nanocomposite gels, further enhance mechanical robustness.

**Figure 3:**
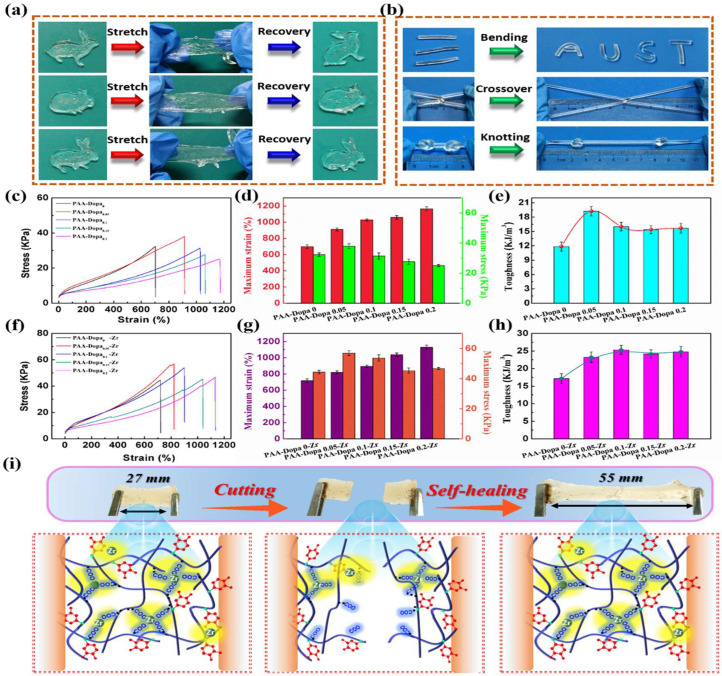
(**a**) Optical images of the PAA-Dopa gels under tensile stretching; (**b**) gels under bending, knotting, and crossing stretching; (**c**,**d**) stress–strain curves, tensile strength, and elongation at break of the gels with different PAA-Dopa content, respectively; (**e**) fracture toughness of different gel samples; (**f**–**h**) behavior of stress–strain curves, tensile strength, elongation at break, and fracture toughness of different gel samples; and (**i**) optical images of the mechanisms of self-healing in the gel samples before and after healing. Reproduced from [55].

Figure 3 illustrates that flexible strain-sensitive sensors are essential components in next-generation applications, including wearable electronics, soft robotics, and human–machine interfaces, where accurate detection of deformation under diverse environmental conditions is required. However, conventional hydrogel-based sensors often suffer from limited mechanical robustness, poor adhesion, and instability under extreme temperatures, which restrict their practical applications. Inspired by the strong adhesive capability of mussels—particularly the role of catechol-containing molecules (e.g., dopamine)—mussel-inspired hydrogels have emerged as a promising class of materials for advanced sensing systems [52]. These hydrogels incorporate dynamic catechol chemistry, enabling strong and reversible interactions such as metal–ligand coordination, hydrogen bonding, and covalent crosslinking. As a result, they exhibit enhanced adhesion, self-healing ability, and tunable mechanical properties. In this context, flexible strain sensors assembled from mussel-inspired hydrogels offer a unique combination of tailored mechanical properties and environmental adaptability. By adjusting the crosslinking density and interaction types, the hydrogel network can be engineered to achieve multifunctional properties, including high stretchability, toughness, and sensitivity, enabling precise detection of both small and large deformations [53]. A key advantage of these systems is their wide temperature tolerance, achieved through strategies such as the incorporation of antifreeze agents, ionic species, or solvent engineering. This enables the hydrogel to maintain mechanical flexibility and stable electrical performance across a broad temperature range, including subzero environments where traditional hydrogels typically fail due to freezing. Finally, the sensing mechanism is generally based on strain-induced changes in ionic or electronic conductivity, where deformation alters the internal conductive pathways, producing measurable electrical signals. Combined with their strong surface adhesion, these hydrogels can form intimate contact with various substrates, such as human skin, ensuring reliable and low-noise signal acquisition even during dynamic motion [54].

### 3.2. Electromechanical Properties

The electromechanical properties describe how their electrical response changes under mechanical deformation, which is fundamental to their sensing performance. These properties arise from the interaction between the deformable polymer network and conductive pathways within the gel.

**Figure 4:**
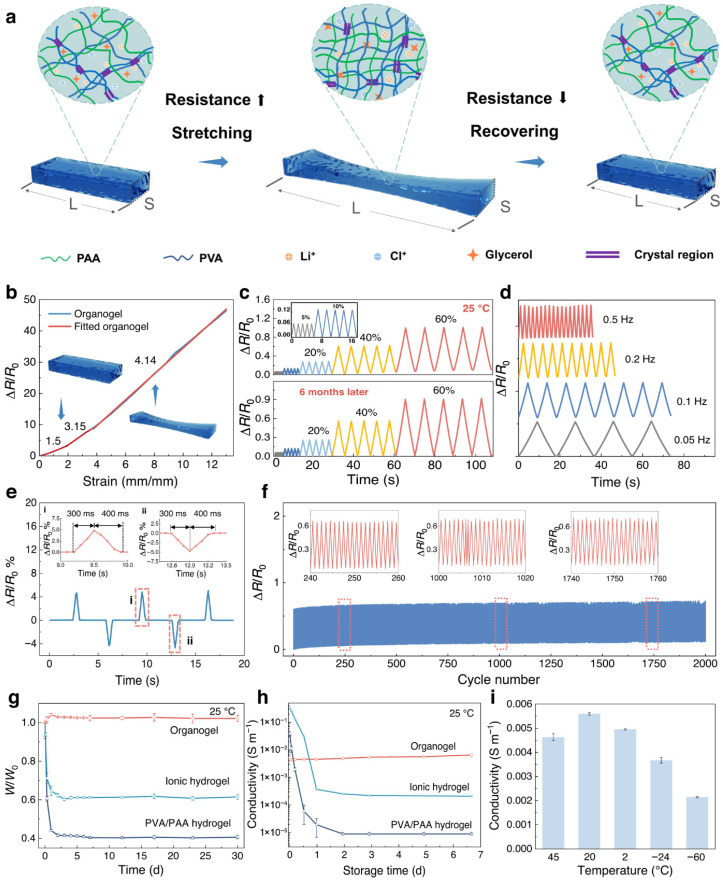
(**a**) The schematic illustration of electromechanical properties through strain sensing during stretching and recovery process; (**b**) behavior of resistance change in the organogel-based sensor against applied mechanical strain; (**c**) relative resistance response of organogel sensor at room temperature under different strains; (**d**) behavior of relative resistance as a function of frequency; (**e**) illustrations of the behavior of response and recovery time; (**f**) electromechanical stability and durability of the sensor for 2000 cycles; (**g**,**h**) change in weight and change in conductivity of organogel, ionic hydrogel, and PVA/PAA hydrogel at room temperature; (**i**) electrical conductivity of organogel at different temperatures. Reproduced from [59].

When the gel is stretched, compressed, or bent, its internal conductive network—formed by ions or conductive fillers is altered. This leads to measurable changes in electrical resistance (piezoresistive effect) or capacitance (capacitive sensing). Another important property is high sensitivity, often quantified by the gauge factor (GF), which indicates how effectively the sensor converts mechanical strain into an electrical signal [56]. Gel-based sensors can exhibit a wide range of sensitivities depending on their composition and structure, with nanocomposite gels typically showing enhanced performance due to improved modulation of the conductive network. Gel-based sensors also exhibit stable and repeatable signal responses under cyclic loading. Their soft and elastic structure allows them to maintain consistent electrical performance during repeated deformation. Figure 4 illustrates that continuous passive motion (CPM) systems are widely used in rehabilitation therapy, assisting joint recovery after surgery or injury by enabling controlled, repetitive movement without active patient effort. A critical challenge in advancing CPM technology lies in integrating soft, durable, and highly responsive sensing materials that can operate reliably under continuous mechanical deformation. Recent developments in ionotronic materials have opened new pathways for creating flexible, skin-compatible sensors [57]. Among these, organogel-based ionotronics have emerged as particularly promising for multifunctional applications due to their nonvolatile nature and resistance to dehydration. These properties contribute to stable performance across a wide temperature range, overcoming key limitations of traditional hydrogels. In this context, the development of tough, rapidly self-recovering, and responsive organogels represents a significant advancement. Such organogels can withstand repeated mechanical stress by employing dynamic polymer networks with reversible physical interactions [58], ensuring low hysteresis, high durability, and consistent sensing performance during long-term cyclic operation.

The ionotronic functionality arises from efficient ion transport within the organogel matrix. Here, mechanical stimuli induce changes in ionic pathways, resulting in measurable electrical signals. This enables real-time monitoring of motion parameters, including joint angle, displacement, and applied force. Such materials are highly suitable for intelligent CPM systems, where continuous feedback is essential for optimizing rehabilitation protocols [60]. By integrating robust mechanical properties, fast recovery, and sensitive electromechanical responses, organogel-based ionotronics enable adaptive, feedback-driven therapy, thereby enhancing patient outcomes and device reliability. The presence of ionic conductivity in many gels (especially hydrogels and ionogels) introduces additional electromechanical behavior. Under deformation, the mobility and distribution of ions change, affecting conductivity and enabling sensing mechanisms based on ion transport and polarization. These sensors often exhibit low hysteresis and fast response/recovery times, which are important for accurately tracking dynamic motions [61]. However, viscoelastic effects in gels can sometimes contribute to minor hysteresis, depending on the material design. Another notable feature is multifunctional responsiveness, where gels can simultaneously respond to multiple stimuli, resulting in coupled electromechanical signals. Well-dispersed conductive fillers help maintain continuous electrical pathways even under large deformations, thereby improving reliability and sensitivity.

### 3.3. Electrical Properties

Electrical properties are fundamental to their ability to detect and convert external stimuli into measurable signals. These properties arise from the presence of mobile ions and/or conductive fillers within the gel’s three-dimensional polymer network. One of the primary characteristics is electrical conductivity, which can be either ionic in hydrogels and ionogels or electronic, enabled by conductive fillers. Ionic conductivity results from the ions within the liquid phase, while electronic conductivity depends on the formation of continuous conductive pathways [62]. The electrical behavior of gels can be adjusted by varying factors such as polymer composition, crosslinking density, solvent content, and filler concentration. This tunability allows optimization for different sensing mechanisms and applications. Gel-based sensors also exhibit piezoresistive and capacitive electrical responses, where resistance or capacitance changes in response to applied mechanical stimuli. These electrical variations form the basis for detecting strain, pressure, and touch.

**Figure 5:**
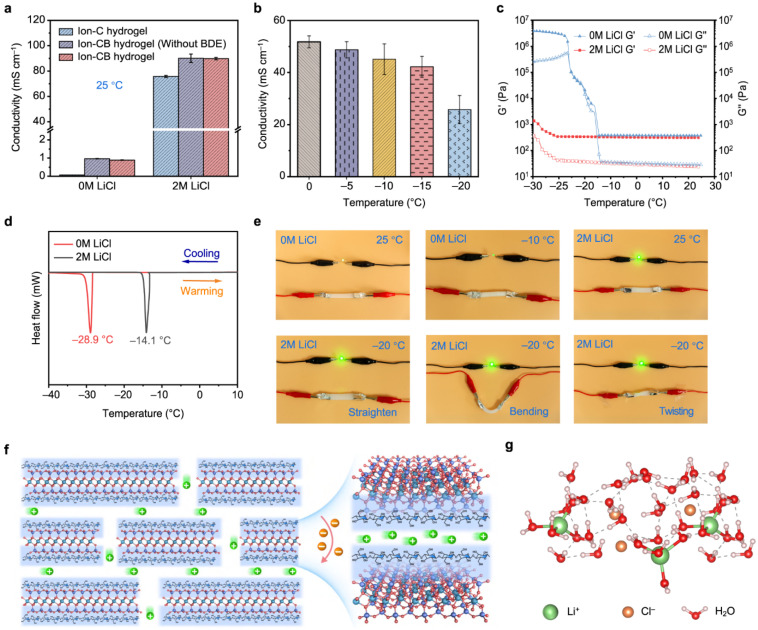
(**a**) Ionic conductivity of the hydrogels at room temperature. (**b**) Behavior of ionic conductivity of ion-CB hydrogels with different temperatures. (**c**,**d**) Storage modulus, loss modulus, and DSC behavior of the hydrogels. (**e**) Different optical images of luminance of LEDs using different hydrogel samples in a flat, bending, and twisting state at different temperatures. (**f**) The mechanism of ionic movement onto the layered BT nanoplatelets with fibers. (**g**) A schematic illustrating freezing tolerance mechanisms of hydrogels. Reproduced from [64].

Figure 5 presents an all-natural hydrogel fabricated from cellulose and bentonite clay, achieving a balance of high mechanical strength, good ionic conductivity, and robust freezing tolerance. The key innovation lies in the coordination interactions between cellulose chains and bentonite nanosheets, forming a robust and multifunctional gel network. In terms of material composition, cellulose serves as a smart, biodegradable natural polymer that provides a flexible and hydrophilic network, whereas bentonite is a clay mineral composed of layered silicates with a high surface area and abundant active sites for interaction with the cellulose matrix [63]. Regarding interfacial interactions, strong coordination bonding between the hydroxyl groups of cellulose and the surfaces of bentonite is proposed. These interactions result in a dense, crosslinked yet dynamic network, ultimately providing a stable material with high structural integrity and flexibility.

From a mechanical perspective, bentonite acts as a reinforcing filler, enabling high mechanical strength. The hydrogel also exhibits controlled energy dissipation and high stretchability despite its high tensile strength. In terms of ionic conductivity, the water content within the hydrogel facilitates ion mobility associated with bentonite [65], resulting in stable ionic transport suitable for flexible electronic sensors. A key feature of such systems is their high dielectric properties, particularly in ion-containing gels, which enhance performance in capacitive sensors. The presence of mobile ions contributes to the formation of an electric double layer (EDL) at interfaces, significantly increasing capacitance. In addition, these gels demonstrate low operating voltage and high energy efficiency, especially in ionic systems where charge transport occurs readily. This makes them well-suited for wearable and battery-free sensing devices. Gel-based electronic sensors often maintain stable electrical performance under deformation, preserving conductivity even when stretched or bent. This is attributed to the flexible polymer network and well-dispersed conductive components [66]. Other notable electrical properties include fast signal response, low noise levels, and good repeatability, all of which are essential for accurate and reliable sensing.

### 3.4. Thermal Properties

Thermal properties play an important role in determining the performance, stability, and reliability of gel-based sensors under varying temperature conditions. These properties are governed by the polymer network structure, solvent content, and conductive components within the gel. One key property is thermal stability, which refers to the ability of the gel to maintain its structural integrity and sensing performance over a range of temperatures. Hydrogels may experience dehydration at elevated temperatures, whereas ionogels and organogels generally exhibit better thermal stability due to the use of low-volatility solvents [67]. Another important aspect is thermal conductivity. Gel-based sensors typically exhibit low to moderate thermal conductivity, as the polymer matrix and liquid phase limit heat transfer. However, the incorporation of conductive fillers such as graphene or carbon nanotubes can enhance heat conduction.

**Figure 6:**
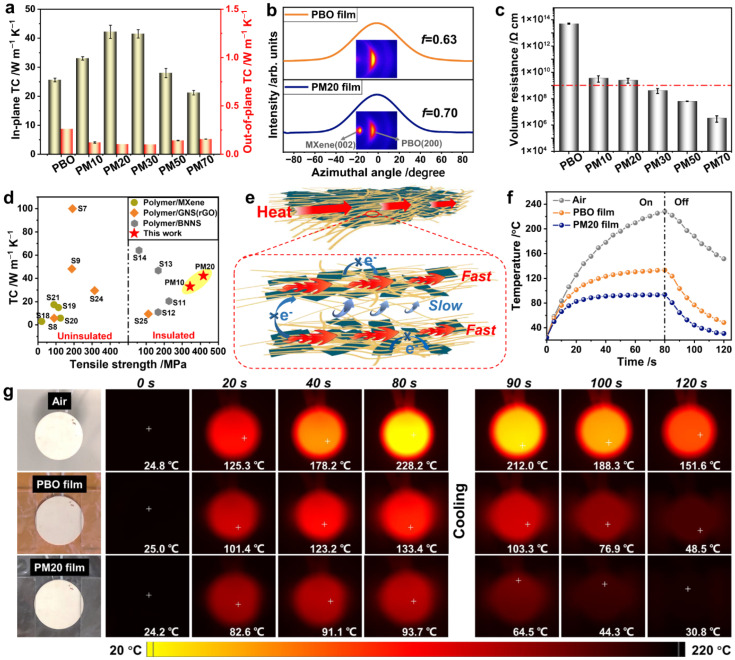
Overview of the thermal properties of the composites: (**a**) In-plane and out-of-plane thermal conductivity behavior of the samples. (**b**) WAXS and azimuthal profiles for the crystal plane (200) reflections of PBO fibers. (**c**) Volume resistance of PBO and MXene hybrid fibers. The red line denotes the critical threshold for insulating resistance. (**d**) Thermal conductivity and tensile strength behavior of films and other polymers or 2D inorganic nanosheet samples. (**e**) Anisotropic behavior of thermal conductivity and electrical insulation of the films. (**f**) Surface temperature–time curves for PBO films to dissipate heat generation. (**g**) IT thermal images of the composites. Reproduced from [69].

Figure 6 presents a high-performance composite film combining poly(p-phenylene benzobisoxazole) (PBO) with MXene nanosheets. This film exhibits a balance of electrical insulation, enhanced thermal conductivity, good mechanical strength, and robust thermal stability. The material design involves the integration of a PBO polymer matrix with MXene nanosheets. The PBO polymer provides high strength, heat resistance, and structural integrity, while MXene contributes to improved thermal conductivity. Although MXene is typically electrically conductive, the composite structure is engineered to maintain electrical insulation while retaining its thermal advantages. From a structural engineering perspective, MXene nanosheets are well aligned within the PBO matrix, and strong interfacial interactions between PBO and MXene promote high mechanical strength [68]. Additionally, the incorporation of MXene acts as a barrier layer, limiting heat and oxygen transfer. It also promotes char layer formation, thereby reducing flame propagation and heat release in the composite films. Electrical insulation is achieved by carefully controlling the dispersion and spacing of MXene, preventing the formation of percolating conductive networks within the composite. Gels also exhibit temperature-dependent electrical behavior, where conductivity varies with temperature due to increased ion mobility or enhanced electron transport. This makes gel-based sensors suitable for temperature sensing applications, as well as for compensating thermal effects in multifunctional devices. The heat capacity of gels is relatively high, especially in hydrogels with high water content, allowing them to absorb and store thermal energy. This contributes to thermal buffering and stability in wearable applications. 

Another critical property is thermal expansion, where gels expand or contract in response to temperature changes [70]. This can influence both mechanical deformation and electrical response, particularly in highly sensitive sensors. Gel-based systems may also exhibit phase transitions, such as gel–sol transitions or volume changes in response to temperature. Thermo-responsive gels can exploit these behaviors for smart sensing and actuation systems. Finally, thermal durability and cycling stability are crucial considerations, as repeated heating and cooling cycles can alter the gel network, leading to changes in mechanical and electrical performance over time.

## 4. Applications

**Figure 7:**
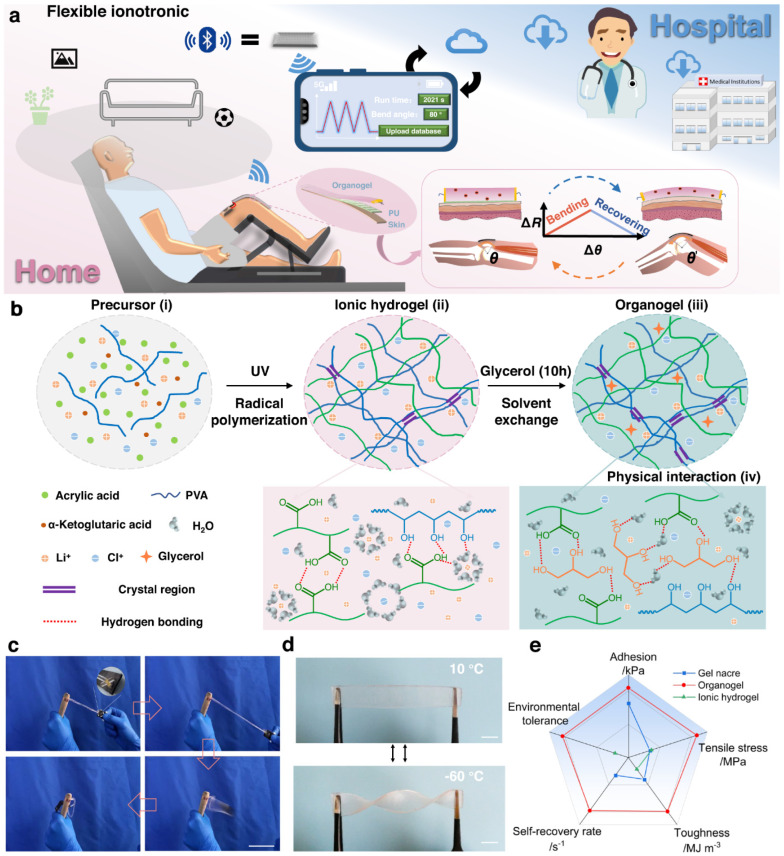
(**a**) Illustration of the intelligent exercise sensing system which involves intelligent CPM equipment useful at home; (**b**) schematic showing preparation process such as (i,ii) free radical polymerization and (iii) solvent exchange system (iv) organogel and physical interactions with in the system; (**c**) optical images showing superior mechanical properties of organogels, with a scale bar of 10 cm; (**d**) images showing anti-freeze behavior of organogels before and after being frozen at −60 °C, with a scale of 1 cm; and (**e**) comparison of different properties of organogels, gel nacre, and ionic hydrogel. Reproduced from [59].

Gel-based electronic sensors are widely used in advanced technologies due to their flexibility, stretchability, biocompatibility, and high sensitivity. Their soft and adaptable nature allows them to function effectively in environments where traditional rigid sensors are unsuitable. One of the most prominent applications is wearable health monitoring systems. Gel-based sensors can be attached to the skin to continuously monitor physiological signals such as heart rate, respiration, body motion, and temperature. Their soft structure ensures comfort and accurate signal acquisition over extended periods [71]. They are also extensively used in electronic skin (e-skin) for robotics and prosthetics. These sensors can mimic human skin by detecting pressure, strain, and touch, enabling robots or prosthetic devices to interact more naturally with their surroundings. In human motion detection, gel-based sensors are integrated into flexible devices to track movements such as joint bending, walking, and facial expressions. This makes them valuable for applications such as rehabilitation monitoring, sports performance tracking, and gesture recognition. Figure 7 presents a tough and highly resilient organogel-based ionotronic device designed for intelligent continuous passive motion (CPM) systems commonly used in rehabilitation therapy. These organogels are fabricated using a polymer network within an organic solvent matrix. The addition of ionic species enables electrical conductivity. Compared with hydrogels, organogels exhibit better resistance to drying, a wider operating temperature range, and higher durability [72]. They also demonstrate high mechanical strength, rapid self-recovery, and robust fatigue resistance.

The system integrates mechanical robustness, rapid self-recovery, and responsive sensing for real-time motion monitoring and feedback. In terms of self-recovery, these organogels rely on dynamic physical interactions, including hydrogen bonding, van der Waals interactions, and reversible network entanglements. After mechanical deformation, the material can rapidly reform its structure [73], resulting in minimal hysteresis losses and long-term durability. These sensors enable real-time motion tracking, feedback-controlled therapy, and intelligent sensing systems. Another key application is in soft robotics, where gel-based sensors provide real-time feedback on deformation, force, and environmental interactions. Their high deformability allows seamless integration with soft actuators. Gel-based sensors are also used in pressure and tactile sensing systems, including touch panels and smart interfaces, where their high sensitivity enables the detection of small forces and high-resolution sensing. In the biomedical field, particularly in tissue engineering, these sensors are applied in implantable and bio-integrated devices due to their biocompatibility and tissue-like mechanical properties [74]. They can monitor internal physiological conditions or assist in drug delivery systems. Additionally, gel-based sensors are utilized in environmental monitoring, where they detect changes in humidity, temperature, and chemical composition. Their responsiveness to external stimuli makes them suitable for smart sensing platforms. They are also being explored in energy harvesting and self-powered systems, where mechanical energy (e.g., motion or pressure) is converted into electrical signals for battery-free operation.

### 4.1. Wearable Health Monitoring Systems

These systems are advanced soft electronic devices that use gel materials to continuously track physiological signals from the human body. These systems are designed to provide real-time, non-invasive, and comfortable monitoring, making them highly suitable for long-term healthcare applications. A key feature of these systems is their excellent skin conformity and softness. The mechanical properties of gels closely match those of human tissue, allowing intimate contact with the skin and reducing motion artifacts. This results in high-quality signal acquisition even during body movement [75]. Gel-based wearable sensors can monitor a wide range of biophysical and biochemical signals, including heart rate, pulse, respiration, body temperature, muscle activity (EMG), and motion. Some advanced systems are also capable of detecting biomarkers in sweat, enabling continuous biochemical analysis. These systems rely on electromechanical and electrochemical sensing mechanisms, where deformation of the gel is critical. Also, their interaction with biological fluids leads to measurable changes in resistance, capacitance, or ionic conductivity [76]. This enables accurate detection of subtle physiological changes. 

**Figure 8:**
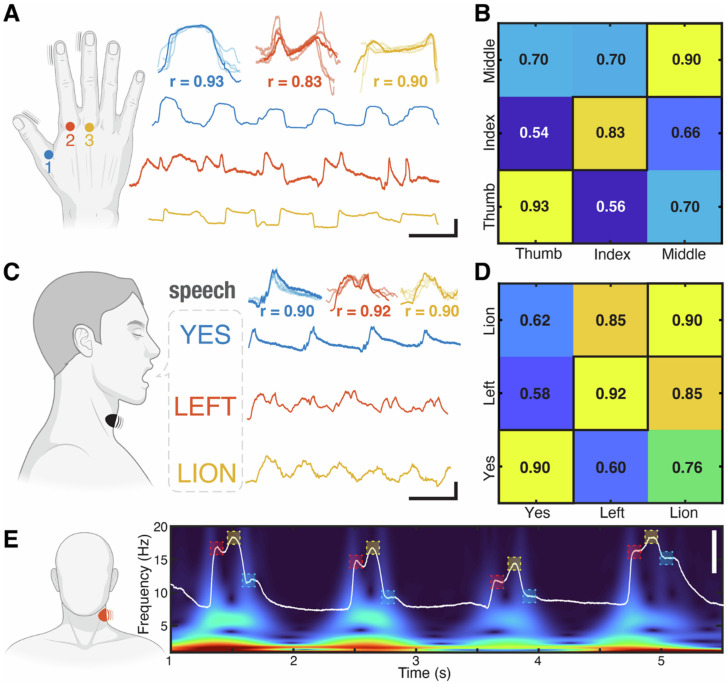
(**A**) MMG recordings demonstrating distinct peaks obtained from piezo-IGTs placed on three fingers, namely the thumb, index, and middle finger. Samples traces of piezo-IGT-acquired thumb movements, index finger movements, and middle finger movements. Scale bar 1 s, 1 mA. (**B**) Cross diagram of three piezo-IGT acquired MMG sample traces, describing the identities in repeated movements across different tests. Each portion of the cell represents the correlation coefficient between two fingers. (**C**) Piezo-IGTs used for speech recognition generate different patterns of spoken words like yes, left, and lion. Scale bar 1 s, 3 mA. (**D**) Cross diagram of piezo-IGT reported through vocal vibrations from spoken words. (**E**) Amplified MCG recordings acquired with piezo-IGT on the neck over the left carotid artery, with corresponding spectrogram. Key features of the carotid waveform include the anacrotic notch, dicrotic notch, and systolic peaks. Scale bar is 2 mA. Reproduced from [77].

Figure 8 presents a hybrid sensing platform that integrates piezoelectric materials with ion-gated organic electrochemical transistors (OECTs). The aim is to achieve highly sensitive vibration detection with built-in signal amplification. This system converts mechanical vibrations directly into amplified electrical signals. The device architecture consists of a piezoelectric layer, ion-gated OECTs, and their coupling mechanisms. The piezoelectric layer generates an electrical potential under mechanical deformation, while the ion-gated OECTs comprise organic semiconductor channels and an electrolyte or ionic medium for gating. The output from the piezoelectric layer acts as the gate voltage for the transistor. The working principle involves piezoelectric conversion combined with ion-gated transistor operation.

Unlike conventional sensors, OECTs provide intrinsic amplification [78], where a small input voltage generated by the piezoelectric effect produces a large output current. This eliminates the need for external amplifiers, reduces noise, and enables the detection of weak signals with high sensitivity. Key features of this system include high sensitivity, low operating voltage, fast response time, and the capability for real-time monitoring. Other important properties include biocompatibility and breathability, which ensure safe and irritation-free contact with the skin over extended periods [79]. Many gel-based systems also exhibit self-healing and stretchability, enhancing durability and reliability under repeated use.

Gel-based wearable devices often support wireless and battery-free operation, integrating with flexible circuits and communication modules for real-time data transmission. This makes them ideal for remote health monitoring and telemedicine. Applications of these systems include continuous patient monitoring, rehabilitation tracking, fitness assessment, infant care, and early disease detection [80]. They are also widely used in personalized healthcare, where individual health data can be continuously collected and analyzed over time. Gel-based sensors offer significant advantages due to their multifunctionality. These advantages are in terms of flexibility, sensitivity, biocompatibility, and light weight. Their wearability with human skin and soft tissue makes them ideal for fabricating wearable devices. These devices are highly suitable for tracking physiological signals such as heartbeat and biochemical markers [81]. One key advantage is their excellent mechanical compliance and stretchability. These properties of gel-based sensors closely matched human skin. Moreover, gel-based sensors exhibit high sensitivity, allowing the detection of even small stimuli like touch or small pressure. Another advantage is their biocompatibility, particularly for hydrogels that contain a high amount of water. This makes them useful for prolonged skin contact without significant irritation. These sensors, based on gels, also exhibit multimodal sensing, such as pressure, strain, or sweat, enabling their use in healthcare activities [82]. Moreover, many hydrogels exhibit self-healing properties, ensuring high durability. However, they have limitations too, which must be addressed before their use in wearable technologies. A major challenge is their poor long-term stability, especially for hydrogels. These hydrogels suffer from water evaporation, dehydration, and swelling in a humid environment. These factors lead to a loss in mechanical and electrical properties, leading to signal drift over time. Another main challenge is their poor mechanical robustness, which limits their use in high-load applications [83]. For example, when these gels are stretched to large strains, they are prone to tearing easily. Moreover, their environmental sensitivity can further degrade their performance. Finally, for particular aspects of performance, their scalability and fabrication still remain challenging. For example, the fabrication process involves controlled curing and complex formulations that are costly. Moreover, biocompatibility may also be challenging depending upon the toxicity of the filler or curatives used during fabrication.

In organogels and hydrogels, the piezo-resistivity originates from a change in internal electrical conductivity. This change arises when the sensor is subjected to mechanical strain. Its simplicity, high sensitivity, and compatibility with soft materials make it particularly suitable for continuous physiological monitoring. At the microscopic scale, several mechanisms contribute to this piezo-resistivity [84]. For example, tunneling effects dominate at low filler content in gels and there are changes in resistance under large deformations. At high filler content, the contact resistance changes, and filler network reconfigurations become the main factor of resistance change. In ionic gels, this deformation can also disturb the ion transport pathways and concentration distributions. This finally results in resistance variations through ionic mobility changes. For wearable health monitoring, such piezo-resistivity behavior enables the detection of a wide range of physiological signals. For example, small external stimuli like pulse waves or muscle activity can result in a change in resistance [85]. The soft nature of gels can ensure intimate skin contact by enhancing signals and reducing motion artifacts. In addition to these advantages in piezo-resistivity mechanisms, some challenges like non-linearity, hysteresis losses, and signal drift need to be solved. These challenges originate from the viscoelastic nature of gels. The humidity and temperature also influence the piezo-resistivity of these gels. Finally, adding stable conductive pathways under large deformations remains a challenge due to mechanical fatigue.

### 4.2. Electronic Skin

Electronic skin refers to flexible, stretchable sensing systems that use gel materials to mimic the structure and functionality of natural human skin. These systems are designed for wearable applications, enabling real-time detection of mechanical and physiological stimuli with high sensitivity and comfort. A key characteristic of gel-based e-skin is its softness and mechanical compatibility with human skin [86]. The low modulus and high stretchability of gels allow the device to conform closely to curved and dynamic body surfaces, ensuring stable contact and accurate signal acquisition during movement. Gel-based e-skin can detect a variety of stimuli, including pressure, strain, temperature, and humidity. These sensing capabilities are achieved through changes in electrical resistance, capacitance, or ionic conductivity when the gel structure is deformed or interacts with environmental factors [87]. Another important feature is high sensitivity and fast response, enabling the detection of subtle signals such as pulse waves, slight pressure changes, or minor body motions. This makes gel-based e-skin highly suitable for health monitoring and human–machine interaction.

**Figure 9:**
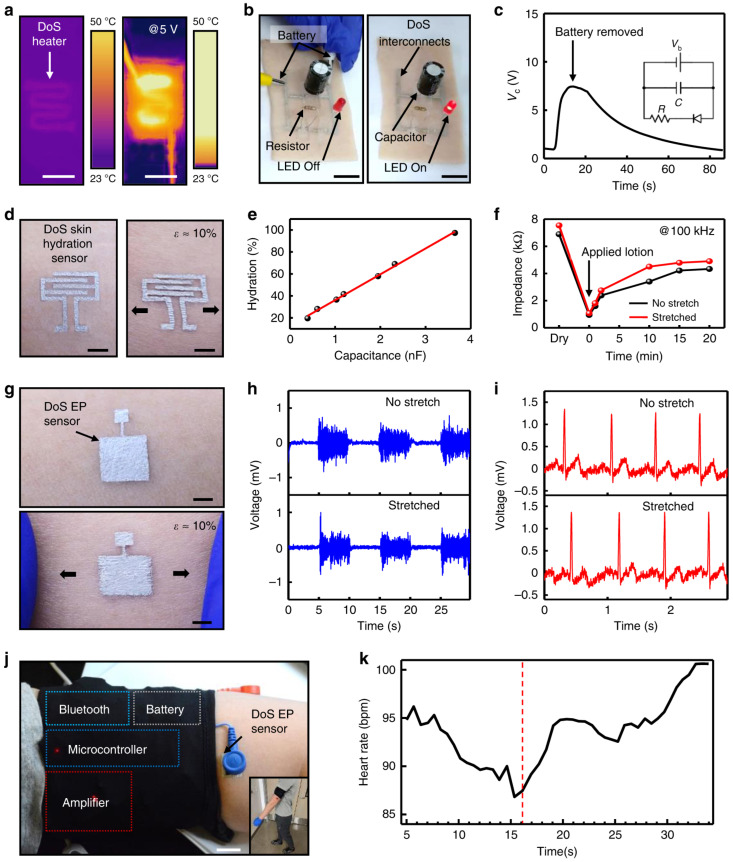
(**a**) Overview of the IR camera of the DoS heater on skin with and without applying a voltage of 5 V. Scale is 1 cm. (**b**) Conducting inks serving as interconnects for charging a capacitor and discharging through a resistor and LED lights. Scale bar is 2 cm. (**c**) Charge–discharge cycle of integrated battery; inset shows the circuit diagram of the electronic component. (**d**) DoS skin with and without applied strain. Scale is 2 mm. (**e**) Calibration of DoS skin hydration sensor through commercial one. (**f**) Impedance of DoS skin hydration with and without mechanical stretch. (**g**) DoS EP sensor integrated on skin with and without stretch. (**h**) Recording EMG signals with and without applied strain. (**i**) Recording ECG signals with and without strain. (**j**) Overview of assembly of Bluetooth, battery, microcontroller and amplifier. Scale is 2 cm. (**k**) Average beats per minute over all trials taken from ECG signals obtained from integrated DoS EP sensor. The area before the red line indicates the subject is standing still, and the area after the red line indicates the behavior when the subject starts walking. Reproduced from [93].

Figure 9 introduces soft, nature-inspired drawn-on-skin electronics (DoS), which are directly written onto the skin using conductive inks. The materials used in their fabrication include conductive inks, elastomeric binders, and biocompatible, skin-safe formulations. These systems provide intimate, conformal contact, enabling high-fidelity sensing and localized therapy without bulky wearable hardware. The key innovation involves the use of conductive inks along with semiconducting or insulating layers [88]. The salient features of these DoS systems include lightweight design, softness, high stretchability, and flexibility. They adhere to the skin through van der Waals forces and mechanical interlocking, and they are often breathable and comfortable for long-term wearable applications. These sensors are multifunctional and capable of detecting signals such as ECG, EMG, EEG, temperature, and strain. Many gel-based e-skin systems also exhibit self-healing, transparency, and biocompatibility, which enhance durability, aesthetics, and safety for long-term use. Their ability to maintain functionality under repeated deformation is crucial for continuous monitoring applications. In addition, these systems can be integrated with flexible electronics and wireless communication modules, enabling real-time data transmission and remote monitoring [89]. Some advanced designs also support multifunctional sensing by combining mechanical, thermal, and biochemical detection within a single platform. Applications of gel-based e-skin include wearable health monitoring, prosthetics, soft robotics, human motion tracking, tactile sensing, and interactive devices. They are particularly valuable for developing next-generation wearable systems that closely replicate the sensory capabilities of human skin.

There are many merits to using gel-based sensors for electronic skin, such as flexibility, self-healing capability, tunable stiffness, and ionic conductivity. Their flexibility allows for comfortable contact between the sensor and human skin. Moreover, various gel-based materials exhibit mechanical robustness, such as high stretchability. This assists and enables them to sustain continuous mechanical deformations [90]. The multimodal sensing ability of these gels, such as strain, pressure, temperature, and humidity, or even biochemical signals, is critical. These features enable gels to perform physiological monitoring effectively within a single platform. Moreover, their ability to tune stiffness and electrical conductivity makes them fit within the interest of the application in need. Moreover, the ionic conductivity in hydrogels allows them to provide stable signal transduction under deformation. These merits are assumed to be highly useful for applications like electronic skins [91]. However, there are various limitations, such as dehydration or swelling of hydrogels, signal drifting, signal decoupling, and data interpretation. Firstly, the hydrogels are prone to dehydration in a dry environment and swelling in the presence of water. This is a critical challenge for their use in electronic skin and leads to performance degradation. The signal drifting, especially under cyclic loadings, can compromise the output signals in terms of accuracy and repeatability. Finally, signal decoupling and data interpretation in multimodal systems are challenging. This is because they require advanced algorithms, often involving machine learning, which adds computational complexity and energy demands [92]. Therefore, addressing these challenges is essential for fully realizing their potential in real-world applications.

### 4.3. Soft Robotics

Soft robotics is an emerging field that utilizes gel materials to design and fabricate soft, flexible robotic systems. These robots are inspired by biological organisms and are characterized by their compliance, adaptability, and safe interaction with humans and delicate objects. A key feature of gel-based soft robots is their high flexibility and stretchability. The soft polymer network allows large deformations, enabling robots to bend, stretch, twist, and compress without mechanical failure [94]. This makes them suitable for navigating complex and constrained environments. Gel materials also exhibit stimuli-responsive behavior, which is essential for actuation. Depending on the design, gels can respond to temperature, pH, electric fields, or moisture, leading to controlled shape changes or motion. For example, electroactive or thermo-responsive gels can expand or contract, enabling soft actuation mechanisms. Another important aspect is the integration of sensing and actuation within the same material system [95]. Gel-based soft robots can incorporate embedded sensing capabilities, allowing them to detect pressure, strain, or environmental changes and respond accordingly, thereby mimicking natural feedback systems.

**Figure 10:**
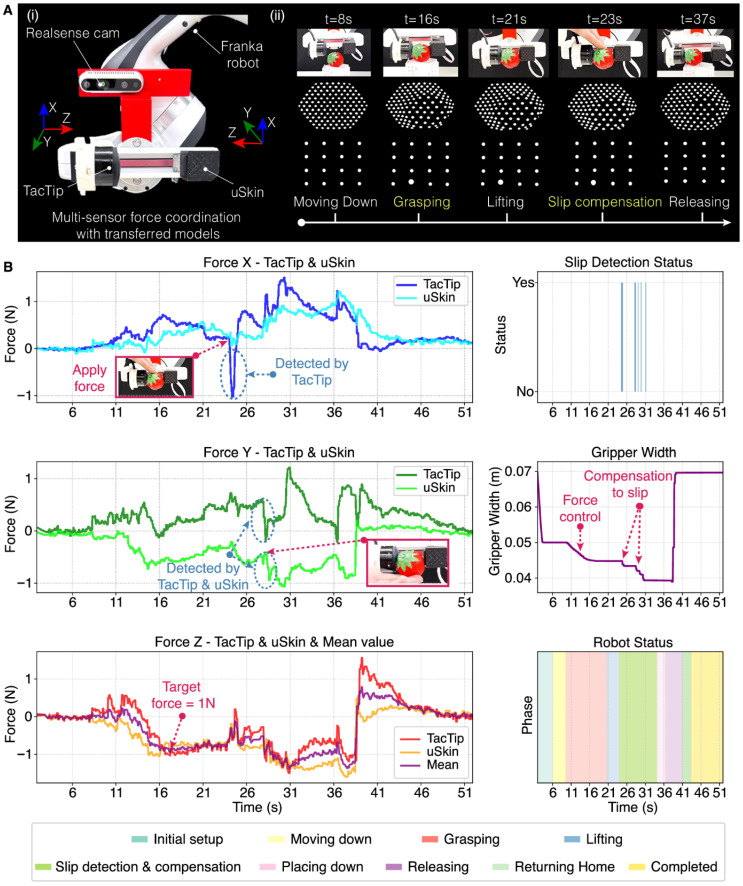
(**A**). Overview of multi-sensor force coordination designed by integrating force models. (**i**) A Franka hand equipped with a Tac Tip sensor on the left and right fingers. (**ii**) Image sequences for robot grasping a strawberry using control, moving down, grasping, lifting, slip detection, and releasing. (**B**). Real-time monitoring of TacTip and uSkin for slip detection status, gripper width, and robot status. Reproduced from [98].

Figure 10 introduces a collaborative learning framework in which multiple tactile sensors share knowledge to improve force-sensing accuracy and generalization. Instead of calibrating each sensor independently, sensors are “trained” using data from one another, similar to model transfer or knowledge distillation in machine learning. The primary motivation is to overcome limitations such as device-to-device variability, non-linear responses, and complex calibration requirements [96], thereby enabling scalable, adaptive, and transferable force sensing across different devices. In this approach, the learning mechanisms are based on supervised learning or transfer learning. The main steps include applying controlled forces to multiple sensors simultaneously, collecting the output signals, and training a model to map these signals to force values. The trained model is then adapted to additional (“student”) sensors using shared datasets. Various types of tactile sensors—such as resistive, capacitive, piezoresistive, and flexible sensors—can be incorporated into this framework [97], supporting applications in electronic skin, human–machine interfaces, and soft sensing systems. Gel-based systems often offer biocompatibility and a water-rich composition, making them ideal for biomedical robotics applications such as minimally invasive devices, drug delivery systems, and artificial muscles. In addition, these robots demonstrate self-healing and durability, as the gel network can recover after damage, thereby enhancing operational lifespan. Their low stiffness also ensures safe human–robot interaction, reducing the risk of injury.

Applications of gel-based soft robotics include grippers, artificial muscles, wearable assistive devices, biomedical implants, and environmental exploration systems [99]. They are particularly well suited for tasks requiring gentle handling, adaptability, and interaction with soft or fragile materials. Gel-based materials enable the robots to help achieve human-like motion and generally good interaction with complex environments. One key merit for their use in soft robotics is their mechanical compliance and stretchability. This soft nature helps them in monitoring contact forces without challenging motions [100]. Similarly, their flexibility helps to adapt the soft robots to perform delicate tasks like gripping fragile and soft objects. Another merit is their high sensitivity and multimodal sensing, which allow them to detect a wide range of stimuli. This enables soft robots to achieve advanced functionalities such as tactile sensing and finally, environmental interactions. In addition to this, many gel-type soft robots have self-healing capacities, allowing fast recovery in dynamic robotic systems. Moreover, their biocompatibility makes them suitable for applications like a wearable robotic system.

### 4.4. Pressure and Tactile Sensing Systems

**Figure 11:**
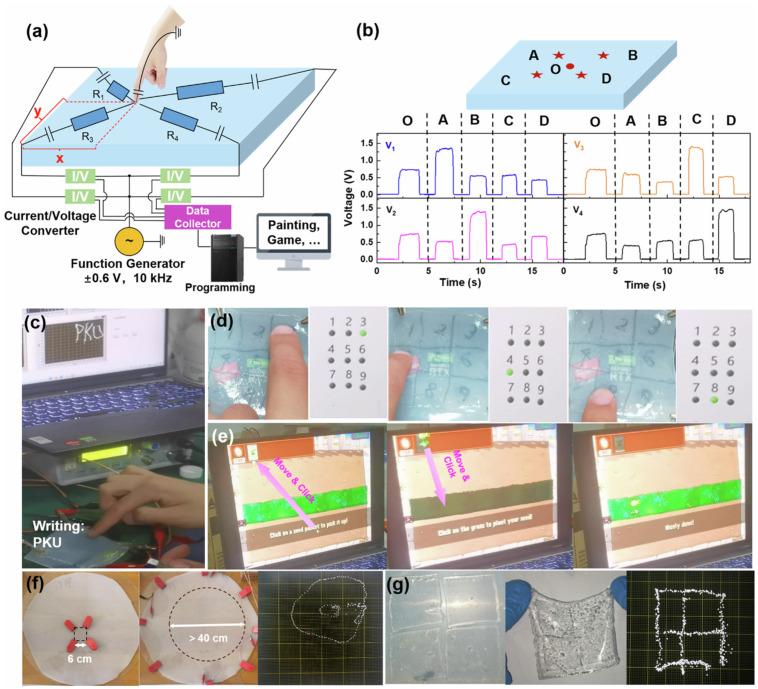
(**a**) Overview of the circuit design of 2D touch panel; (**b**) electrical signal as output voltage from touching different points of the panel; (**c**) pattern painting; (**d**) keyboard touching; (**e**) mouse control for applications like video games for people; (**f**) after reaching a 3500% areal stretching; and (**g**) after self-healing from the sample. Reproduced from [103].

Gel-based pressure and tactile sensing systems are flexible sensing devices that use gel materials to detect and respond to mechanical stimuli like pressure, touch, strain, and deformation. These systems are widely used in wearable electronics, electronic skin, and soft robotics due to their softness, high sensitivity, and excellent mechanical adaptability. A key feature of these sensors is their high sensitivity to small mechanical forces. When external deformation is applied, the internal polymer network of the gel deforms, leading to changes in electrical resistance (piezoresistive), capacitance (capacitive), or ionic conductivity [101]. These electrical variations enable precise detection of both subtle and large inputs. Gel-based tactile sensors also exhibit a wide sensing range and fast response time, allowing them to detect dynamic and static forces effectively. Their soft and deformable nature ensures conformal contact with irregular surfaces, improving signal accuracy and spatial resolution. Another important property is high flexibility and stretchability, which allows the sensors to operate under repeated bending, stretching, and compression without performance degradation. This makes them suitable for integration into wearable devices and artificial skin systems [102]. Many gel-based tactile sensing systems also demonstrate multifunctional sensing capabilities, where they can simultaneously detect pressure, strain, temperature, and humidity. This multifunctionality enhances their applicability in complex sensing environments. Figure 11 presents a dual-crosslinked hydrogel that combines hydrophobic associations and electrostatic interactions to achieve ultrahigh stretchability, self-healing capability, and multifunctional sensing. This material is designed for applications such as flexible touch panels and wearable sensors. The dual-crosslinking strategy involves both hydrophobic and electrostatic crosslinking mechanisms. In hydrophobic crosslinking, hydrophobic domains aggregate to form physical junctions that act as energy dissipation centers and reversible sacrificial bonds. This mechanism provides high toughness and resistance to mechanical failure.

In electrostatic crosslinking, oppositely charged polymer chains interact through ionic bonding, enabling dynamic bond breaking and reformation, as well as relatively fast structural recovery. As a result, the hydrogel exhibits excellent mechanical properties, including high stretchability, strong self-healing capability, and enhanced toughness [104]. In terms of self-healing, mechanical damage breaks reversible bonds, including both hydrophobic and ionic interactions. Upon recontact, the hydrophobic domains reassemble, and the electrostatic interactions reform. This process enables rapid and repeatable self-healing, making the material highly resistant to fatigue under cyclic mechanical deformation.

For flexible touch panel applications, these hydrogels function as conductive sensing layers. They exhibit high sensitivity to external stimuli and can detect multipoint touch [105]. They are also considered multifunctional materials, capable of simultaneously sensing strain, pressure, and touch position. In addition, these systems often exhibit self-healing ability, transparency, and biocompatibility, which enhance durability, usability, and compatibility with human skin. Their ability to maintain stable performance under cyclic loading is critical for long-term applications. Applications of gel-based pressure and tactile sensors include electronic skin (e-skin), human motion detection, robotic grippers, prosthetics, touch interfaces, and healthcare monitoring systems [106]. These materials are particularly valuable for enabling human–machine interaction and real-time feedback systems. These sensors are frequently useful for applications like human–machine interfaces and health monitoring. These gel-based sensors exhibit various merits due to their easy-to-use and multifunctional nature. Their excellent mechanical compliance enhances signal fidelity, which is critical for tactile sensing [107]. These sensors also exhibit high sensitivity and a wide detection range that responds well to both small and large deformations. This makes them suitable for detecting fine tactile features such as texture, vibration, and force distribution. These tactile sensors are also designed to monitor multimodal sensing, thereby allowing simultaneous detection of multiple factors. These factors are pressure, strain, temperature, and humidity, which are useful for advanced electronic skin applications. Despite these many merits, there are challenges too, such as low durability, low mechanical strength, poor filler dispersion, and poor interfaces [108]. As gels are highly stretchable, they exhibit poor mechanical strength and can be fractured or degraded under cyclic mechanical deformations. Moreover, their viscoelastic nature leads to time-dependent responses that cause low responses, delayed recoveries, and baseline drift. This affects the accuracy and repeatability of pressure measurements, particularly in dynamic or cyclic loading conditions. Finally, the spatial resolution and integration challenge also arises in tactile sensing systems. Achieving high-density sensor arrays is difficult due to the challenging fabrication process. The use of additional sensors or electronic components while maintaining flexibility is complicated [109]. Scalability and manufacturing are also costly, resulting in limited synthesis and production at a large scale.

### 4.5. Tissue Engineering and Biocompatibility

Gel-based tissue engineering involves the use of gel materials, primarily hydrogels, as scaffolds to support the growth, proliferation, and regeneration of biological tissues. These gels mimic the natural extracellular matrix (ECM), providing a suitable environment for cells to attach, grow, and differentiate. A key advantage of gels in tissue engineering is their high water content and biocompatibility, which closely resemble natural biological tissues. This enables effective nutrient transport, waste removal, and cell signaling, all of which are essential for tissue development [110]. Gel scaffolds provide a three-dimensional porous structure that allows cells to be encapsulated or seeded within the matrix. This structure supports cell adhesion, migration, and proliferation, facilitating the formation of new tissue. The porosity and mechanical properties of gels can be tailored to match specific tissue requirements, such as soft tissues (e.g., skin and brain) or more structured tissues (e.g., cartilage). Another important property is biodegradability, whereby the gel gradually degrades as new tissue forms, eliminating the need for surgical removal. The degradation rate can be controlled to match the rate of tissue regeneration [111]. Many gel systems are also stimuli-responsive and biofunctionalized, meaning they can incorporate growth factors, drugs, or other bioactive molecules to enhance tissue regeneration. Advanced gels can respond to environmental conditions such as pH, temperature, or enzymatic activity, enabling the controlled release of therapeutic agents. In addition, gels can be fabricated using advanced techniques such as 3D bioprinting, which allows precise control over scaffold architecture and enables the creation of complex tissue structures. Applications of gel-based tissue engineering include skin regeneration, wound healing, cartilage repair, bone tissue engineering, and organ regeneration. These materials are also widely used in drug delivery systems and regenerative medicine. 

**Figure 12:**
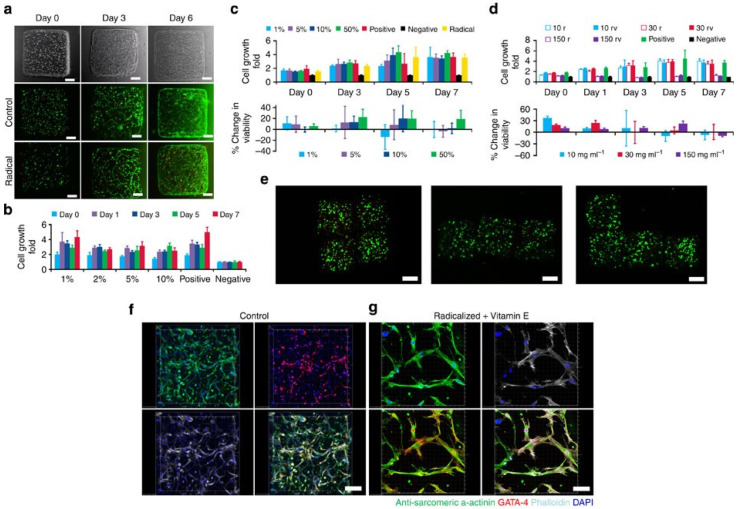
(**a**) Evaluation of cell viability at 0, 3, and 6 days for the control sample (top) and for radical groups soaked into 30 mg/mL for 30 min (bottom). Green profiles represent live cells while red profiles represent dead cells. (**b**) Biological assay results as a function of volume to volume ratio for added radical suspension volume to cell suspension volume for days 0, 1, 3, 5, and 7. All of these final results were normalized with respect to the negative control sample. (**c**) After paramagnetizing hydrogels with radicals, exposure to vitamin E solution improves cell proliferation. (**d**) Later, paramagnetization of hydrogels at radical content of 1,030,150 mg/mL and exposure to vitamin E. Results show that when a large content of radicals is added, like 150 mg/mL, with a 30 min incubation time, vitamin E does not show any significant benefit in recovering cells. (**e**) Fluorescence image of rod-shape, square and L-shaped assemblies of hydrogels. (**f**) Control sample with no radicals at scale of 200 um. (**g**) Image with radical and vitamin E-exposed gels with a scale bar of 100 µm. All images were reported on 10 day. Reproduced from [112].

Figure 12 focuses on magnetoceptive gels embedded with magnetic particles that can self-assemble and reconfigure under external magnetic fields. The study highlights how guided magnetic fields enable precise control over structure, properties, and functionality. These magnetoceptive gels are composed of a polymeric gel matrix and magnetic fillers. Their key features are a soft, deformable structure and their tunable mechanical and electrical properties. They also exhibit a self-assembly mechanism that is reversible under a magnetic field. For example, embedded magnetic particles experience dipole–dipole interactions.

Moreover, they demonstrate alignment into chains, columns, and networks under magnetic fields [113]. This self-assembly feature also enables the precise positioning of particles. In addition, they exhibit the formation of a complex architecture and local tuning of mechanical stiffness under magnetic fields. These materials are multifunctional as they enable shape morphing or their reconfigurations. They also exhibit magnetically driven actuation, adaptive stiffness, and self-healing prospects. Finally, they are useful for various applications like soft robotics and flexible sensors. Hydrogels and organogels are very attractive for tissue engineering and biocompatible interface applications. This is primarily due to their close mimicking of the physical and biochemical properties of native body tissues. For example, their soft, hydrated, and tunable nature allows them to achieve great compatibility with biological tissues. They are further useful for wound monitoring, implantable sensing, and regenerative medicine. They have various merits like biocompatibility, tissue-like properties, breathability, and flexibility [114]. For example, hydrogels are hydrated and have tissue-like extracellular matrices. These features promote cell adhesion, proliferation, and differentiation. These features also make them ideal for interfacing with skin, muscle, and even internal tissues. Another merit is their breathability, which allows the diffusion of oxygen, nutrients, and biochemical signals. This is critical for maintaining healthy tissue environments in both wearable and implantable applications. Moreover, their flexibility and conformability further enhance their comfort and reduce mechanical mismatch, and minimize inflammation. Despite having so many merits, they also have challenges like cytotoxicity, material safety, and biodegradation control. Many hydrogels are prone to degradation, swelling, or dehydration over time, thereby limiting sensing performance [115]. Moreover, matching the degradation rate to tissue regeneration timelines remains complex. For cytotoxicity, the addition of toxic filler and curing agents limits the biocompatibility of these hydrogels. Therefore, safe long-term interactions with biological tissues require the use of medical-grade materials and their validation before implanting.

### 4.6. Environmental Monitoring

Gel-based environmental monitoring systems utilize gel materials as sensitive platforms for detecting and responding to environmental changes. These systems are valued for their high sensitivity, flexibility, and ability to interact with various chemical and physical stimuli. A key feature of gel-based sensors is their stimuli-responsive behavior. Gels can undergo changes in volume, shape, conductivity, color, or mechanical properties when exposed to environmental factors such as temperature, humidity, pH, light, and chemical pollutants [116]. These changes can be readily measured and correlated with environmental conditions. Gel-based systems are widely used for humidity and temperature sensing, where the absorption or desorption of water within the gel affects its electrical or mechanical properties [117]. Similarly, pH-sensitive gels can detect changes in acidity or alkalinity through swelling behavior or variations in conductivity.

**Figure 13:**
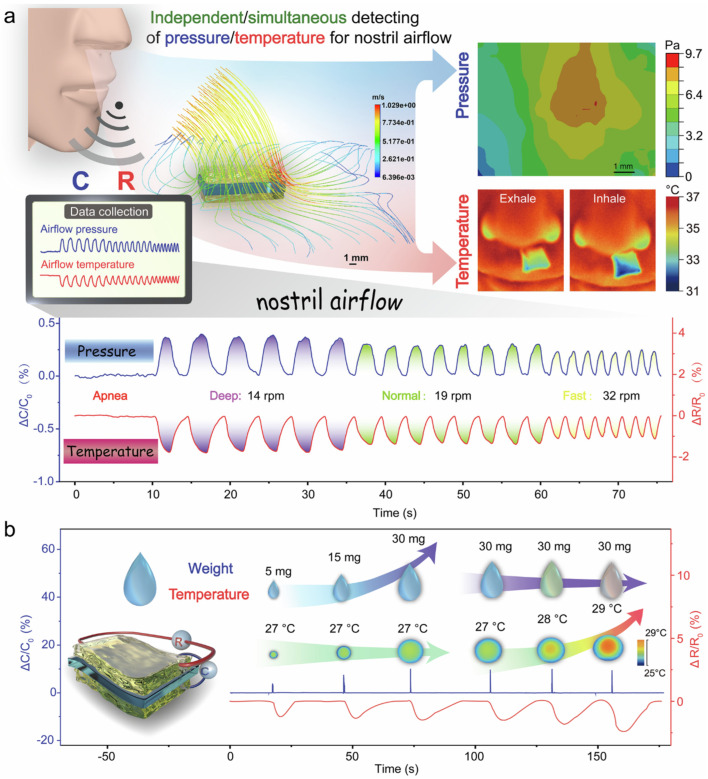
(**a**) Schematic illustration of nostril airflow sensors for temperature and pressure changes during breathing. Thermal change was monitored through an infrared camera while the pressure change was simulated. (**b**) The sensing monitoring for testing water droplets by dual mode, by changing the weight and temperature of the water droplet. Reproduced from [120].

Figure 13 presents a crosslinked eutectogel-based sensor designed for highly sensitive detection of nostril airflow, enabling simultaneous monitoring of respiration pressure and temperature variations. This system is particularly suited for wearable and biomedical respiratory monitoring. A eutectogel is formed by immobilizing a deep eutectic solvent (DES) within a polymer network. Its key features include high ionic conductivity, good thermal conductivity, and low volatility compared to hydrogels. Crosslinking enhances its mechanical strength, structural stability, and durability [118]. The eutectogel structure consists of a chemically crosslinked polymer network that provides elasticity, along with a deep eutectic solvent that serves as the ionic medium. It is soft, flexible, porous, and comfortable for contact with human skin, while also maintaining stability under varying environmental conditions. The sensing mechanism is based on airflow from the nostrils, which induces mechanical deformation of the eutectogel. This deformation alters ion transport pathways, resulting in changes in electrical resistance. As a result, the system can detect breathing rate, airflow intensity, and respiration patterns [119]. This technology is useful for applications such as respiratory health monitoring and smart masks for medical diagnostics.

Another important application of gel-based systems is in chemical and pollutant detection. Functionalized gels can selectively interact with heavy metals, toxic gases, or organic contaminants, leading to measurable changes in electrical or optical signals. This makes them suitable for water quality monitoring and air pollution detection. Gels can also enable colorimetric sensing, where visible color changes occur in response to environmental stimuli, allowing simple and low-cost detection without complex instrumentation [121]. In addition, gel-based sensors offer high flexibility and adaptability, making them suitable for deployment in diverse environments, including wearable environmental sensors, portable devices, and remote monitoring systems. Some gel systems also exhibit self-healing capabilities and long-term stability, enhancing their durability under harsh or fluctuating environmental conditions. Their tunable properties allow customization for specific sensing targets and sensitivity ranges. Applications of gel-based environmental monitoring systems include air and water quality assessment [122], humidity sensing, temperature monitoring, detection of hazardous chemicals, and smart agriculture systems.

One of the key merits of gel-based sensors for environmental monitoring is their high sensitivity and responsiveness to environmental changes. This is due to porous and hydrated networks that facilitate rapid diffusion, enabling fast detection. This behavior makes them promising tools for monitoring environmental factors like gases, vapors, or ions. Gel sensors also offer the tuning of chemical functionality, allowing the incorporation of specific receptors [123]. These receptors are dyes or nanomaterials that help detect heavy metals or hazardous gases in the environment. Moreover, these gel sensors offer low toxicity, making them safe for electronic skin applications. Moreover, most of these gel sensors are recyclable, thereby enhancing sustainability and device durability. Despite having so many advantages, these sensors suffer some limitations. These are cross-sensitivity, limited power supply, scalability, and data interpretation complexity. For example, the sensor response is influenced by various factors, such as humidity, affecting accurate gas sensing activity. To overcome this, effective decoupling of signals remains a solution, but it is challenging too. Moreover, the functioning of a wearable sensor needs a continuous power supply to function, which is difficult for long-term operations, especially in remote areas. In addition, the fabrication process of gels is difficult due to the sensitivity of their properties related to the synthesis method [124]. Finally, data complexity arises in multimodal sensing systems that require advanced and costly optimization tools for optimum functioning.

### 4.7. Energy Harvesting and Self-Powered Systems

Gel-based energy harvesting and self-powered systems are advanced soft electronic platforms. This platform uses gel materials to convert ambient mechanical, thermal, or chemical energy into electrical energy. These systems enable battery-free operation, making them highly suitable for wearable electronics, biomedical devices, and remote sensing applications. A key mechanism in gel-based energy harvesting is the conversion of mechanical energy (e.g., motion or deformation) into electrical signals. This is commonly achieved through triboelectric, piezoelectric, or piezoresistive effects [125]. These mechanisms include deformation of the gel, which alters charge distribution or conductive pathways, generating electrical output. Another important mechanism involves ionic transport and electrochemical processes. In ion-containing gels, movement of ions under mechanical stress or concentration gradients can generate streaming potentials or ionic currents, contributing to energy generation.

**Figure 14:**
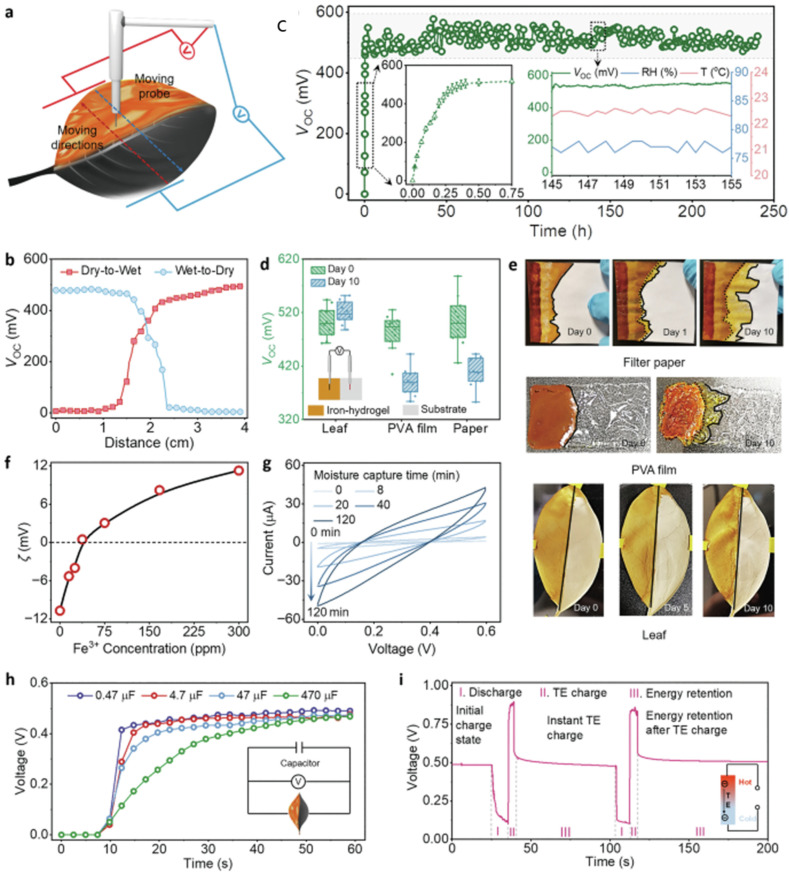
(**a**) Schematic description of monitoring energy harvesting. (**b**) Illustration of monitoring output voltage generation change with change in dry to wet conditions. (**c**) Output voltage under normal, relative humidity change, and different temperatures. (**d**) Change in output voltage with change in substrate. Both papers and PVA film without leaf vein exhibited a loss in voltage after 10 days. This highlights the importance of water movement across the substrate. (**e**) Optical images of water movement across the substrate. (**f**) Zeta potential of the CB with respect to change in iron concentration. (**g**) Behavior of current-voltage curves at a scan rate of 0.05 V/s at different moisture changes in the leaf substrate. (**h**) Discharge of LEH by capacitors with different capacitances. (**i**) Charging behavior of LEH as a potential candidate thermoelectric panel and in a low-grade heat harvesting and storage application. Reproduced from [127].

The approach shown in Figure 14 mimics the structure and function of natural leaves to create a self-sustained energy system capable of both harvesting and storing energy. It utilizes a hygroscopic (moisture-absorbing) iron-based hydrogel that continuously interacts with environmental humidity to generate power. The working principle is based on a hydrogel that absorbs water vapor from the air due to its hygroscopic nature. This moisture absorption and desorption process creates ion concentration gradients [126]. In addition, water diffusion induces swelling and shrinking of the hydrogel system. The design is inspired by natural leaves, which regulate water transport through vein-like channels and maintain continuous transpiration cycles. These systems feature porous structures, gradient distributions, and large surface areas. The porous structure enables efficient moisture exchange, while the large surface area enhances water interaction. The hygroscopic iron-based hydrogel also facilitates redox reactions and improves ionic conductivity.

Gel-based systems are also widely used in triboelectric nanogenerators (TENGs) and ionic thermoelectric devices, where temperature differences or contact electrification generate electrical energy. The high flexibility and deformability of gels increase the effective contact area, thereby improving energy conversion efficiency. One of the major advantages of gel-based energy systems is their high flexibility and stretchability, which allows them to harvest energy from natural human motions such as walking, bending, or breathing [128]. Their soft and skin-compatible nature makes them ideal for integration into wearable devices. These systems also enable self-powered sensing, where the generated electrical signals can be directly used for sensing without requiring an external power source. This reduces system complexity and enhances portability. Additionally, gel-based energy devices often exhibit low operating voltage, lightweight structures, and strong environmental adaptability. Some systems also incorporate self-healing properties and high durability, ensuring stable performance under repeated mechanical stress [129]. Applications include wearable energy harvesters, self-powered sensors, and electronic skin.

Energy autonomy is critical for using gel-based sensors for applications like electronic skin or environment monitoring. Portable batteries are often used to power devices, but this is regarded as a non-sustainable energy source. However, the self-powered systems are developed to supply low-voltage sensors through bio-mechanical energy harvesting [130]. This harvesting concept relies on developing a small power source from mechanical motions like pressing, running, or tapping, etc. Thus, gel-based sensors also offer good mechanical compliance that allows an energy-efficient conversion from natural motions. Another merit is their multifunctional integration in which the gel matrix can serve both as a sensing element and an energy harvesting system. For instance, ionic conductive gels can simultaneously generate electrical signals under deformation while detecting strain or pressure [131]. However, there are some limitations for these energy harvesting systems which must be addressed before large-scale production can proceed. These limitations are low power generation, environmental sensitivity, poor coupling coefficients, and efficient energy storage problems. The most important challenge of low power output limits them to small-scale energy harvesting devices with basic functionalities. Moreover, their environmental sensitivities resulted in poor mechanical-to-electrical coupling efficiencies. These coupling efficiencies also arise due to viscoelasticity, hysteresis, or incomplete recoveries of the structure of gel-based sensors. Finally, energy storage and management remain a challenging task due to non-uniform energy harvesting. Moreover, the harvesting of energy requires efficient energy storage and power regulating circuits that affect their wearability.

### 4.8. Photo-Actuator

**Figure 15:**
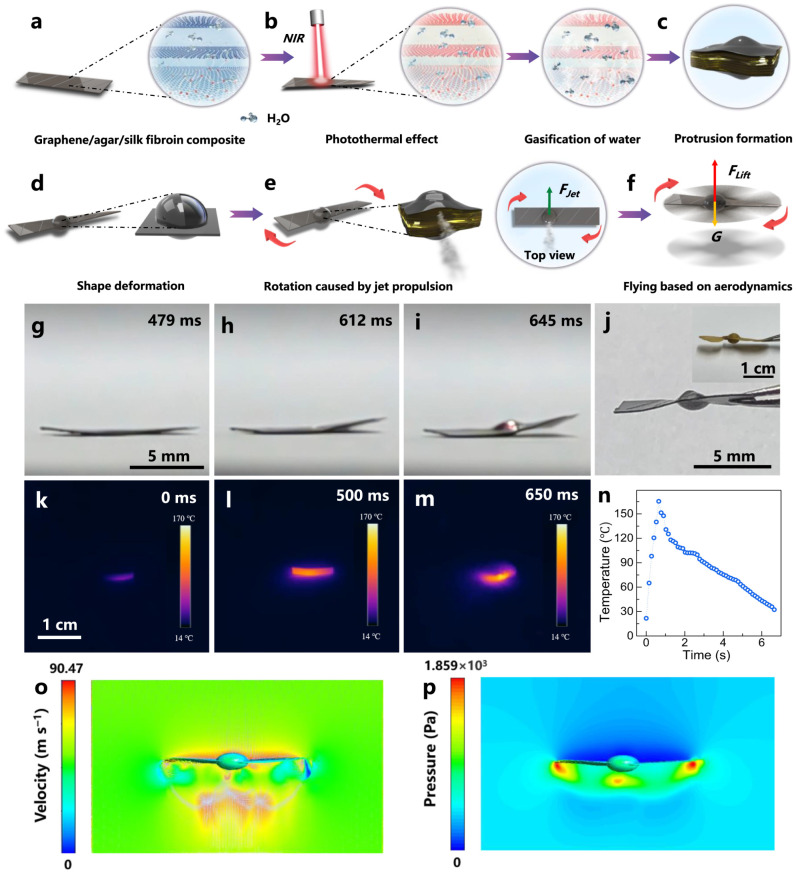
(**a**–**f**) Schematic presenting the flight mechanisms of a photo-actuator, like a helicopter mode; (**g**–**i**) optical images of a high-speed camera integrated with the photo-actuator; (**j**) optical image of airscrew structure; (**k**–**m**) thermal infrared images; (**n**) the relative change in temperature of the photo-actuator; and (**o**,**p**) velocity and pressure field distribution of high-speed photo-actuator with airscrew structures. Reproduced from [134].

Photo-actuators in gel-based sensors are advanced smart systems that convert light into mechanical motion through various mechanisms. These mechanisms are photothermal, photochemical, or photomechanical. In photothermal mechanisms, the light is absorbed, and it is converted into heat. The increase in temperature causes gel swelling or deswelling, and the gel’s key features are a fast response and suitability for remote actuations. In a photochemical reaction, the light induces molecular changes such as bond breaking or the formation of new bonds [132]. These processes result in increased internal stress and deformations. Similarly, the photomechanical effect includes the direct conversion of light into mechanical strain without heating or cooling. This phenomenon is common in liquid crystal elastomer-based gels. Photo-actuators operate by integrating photo-responsive materials into gels. When exposed to light (UV, visible, or near-infrared), the gel changes in its dimensions, shape, or size. The additional changes are expansion or contraction in volume, bending or folding, and surface deformations. The key properties of these photo-actuators include their ability to be controlled remotely, as no physical contact is required. They also exhibit fast response times, high sensitivity to light, and a soft, highly flexible nature [133]. These systems offer several advantages, such as precise spatial and temporal control, energy efficiency, and compatibility with flexible electronics. Their flexibility, remote operation, and responsiveness make them highly promising for next-generation applications, including wearable devices, soft robotics, and biomedical engineering. However, several challenges remain, including the limited penetration depth of light and material fatigue under prolonged cyclic mechanical deformation. Additional limitations include slower responses in thick gels and potential photodegradation of materials. Similarly, Figure 15 illustrates bioinspired rotary flight systems based on light-driven composite films. This approach represents an emerging strategy for wireless, untethered actuation and sensing in gel-based devices. These systems mimic natural flyers, such as seeds, insects, and microorganisms, which achieve rotation and lift through asymmetric structures.

The working principle typically involves gel-based composite films integrated with photoresponsive materials [135], such as graphene derivatives, liquid crystal elastomers, or photothermal nanoparticles. Upon exposure to light, these materials undergo photothermal conversion or asymmetric deformation (e.g., bending or twisting). In terms of material design, these systems consist of soft gel matrices—such as hydrogels, organogels, or elastomers—modified with light-responsive fillers like gold nanorods or other photothermal agents. These materials exhibit desirable properties, including high flexibility, low density, rapid response, and strong sensitivity to light. Their motion can be precisely tuned by adjusting light intensity, film geometry, and environmental conditions such as humidity [136]. These smart materials are highly promising for applications in self-powered sensing platforms and rotary motion-based signal modulation.

There are various merits to using photo-actuation based on gel-based sensors. These include wireless and remote controllability, high spatial resolution, multifunctionality, and energy efficiency. The most promising factor is its ability to function with wireless and remotely controlled technology. This is because the light can be focused and modulated with good accuracy [137]. Another key merit is its multifunctionality, as photo-actuators can simultaneously sense and actuate as needed. For example, a gel can deform under light while also generating an electrical signal, enabling closed-loop systems. Gel-based actuators also exhibit energy-efficient conversion efficiency and are thus sustainable. This is particularly useful while using photothermal conversions, as light is converted into heat. However, there are some challenges to scalable productivity, such as limited light penetration depth, response efficiency, and structural degradation under light [138]. A major challenge is limited light penetration, especially in biological tissues. This restricts the effectiveness in creating biocompatible or implantable applications. They also exhibit weak response efficiency and actuation force. This is due to the restriction of their use in load-bearing or high-force applications. Finally, photodegradation can also occur over repeated light exposure cycles. This results in degradation of photochromic molecules like azobenzene, which results in poor output performance.

### 4.9. Signal Decoupling in Multimodal Sensors

The signal decoupling in sensors refers to their ability to distinguish different signals under mechanical stimuli. This is quite critical for various applications like electronic skin and soft robotics. In gel-based sensors, the multimodal challenge is enabled due to the diverse filler functionalities. These sensors often suffer from signal cross-talk, in which various stimuli produce mixed electrical signals. This process makes the researcher confused about attributing the signal of a specific output. Therefore, the signal decoupling strategies are critical to ensure accurate and reliable data [139]. Various pathways can develop the effective signal decoupling process, such as the selection of the right material and smart structural engineering of the sensor. For instance, the addition of piezoelectric fillers with ionic conductive pathways results in distinct conductive mechanisms. Similarly, structural engineering, like multilayer or heterogeneous constituents, can provide specific responses to a stimulus. Similarly, the use and integration of multi-parameter signals can be helpful to obtain simultaneous monitored data. As each constituent affects the signal in a different electrical response, the models can be used to decouple signals. For example, strain may predominantly affect resistance, while temperature may significantly alter ionic conductivity and thus capacitance. It is widely acknowledged that advances in signal processing through machine learning can help in the decoupling process [140]. This process is based on data-driven models that can understand the non-linear relations between signal and sensor output. The algorithms, such as support vector machines and neural networks, are critical to quantify multiple stimuli with high accuracy. These algorithms are further useful to separate the presence of noise or drift from output signals. In spite of these many procedures for decoupling signals, there are various challenges. These challenges are achieving high durability, minimizing hysteresis losses, and developing standards and protocols. Future technology will rely on edge AI for real-world applications to decouple multiple sensory outputs.

The signal decoupling can be obtained by learning complex patterns in the sensor’s output data. Here, the most widely used approach is employing Convolutional Neural Networks (CNNs) and Recurrent Neural Networks (RNNs). Both of these tools offer distinct advantages depending on the origin of the output signals. The CNN-based signal decoupling is particularly more effective when the output signals represent structural data [141]. These are time–frequency maps, spatial electrode arrays, or multi-channel signal matrices. So, for gel-based sensors, where multiple stimuli may influence the electrical output, the CNN is more efficient. These output data are resistance, capacitance, or output voltage. Here, the CNN can identify subtle spatial patterns that are linked to the specific stimuli. Therefore, the CNN tools are efficient and well-suited for real-time and edge device decoupling in wearable sensors. Similarly, the RNN-based signal decoupling is attributed to handling sequential and time-dependent data. This makes this tool ideal for sensors where stimuli exhibit dynamic behaviors. This tool enables the system to differentiate stimuli based on response evolution over time [142]. For example, mechanical deformation often produces rapid, transient signals, while thermal or humidity effects show slower drift. In such cases, RNNs can decouple these temporary signatures and provide the desired decoupled data. This process makes RNNs valuable in applications like fitness training that involve continuous monitoring.

### 4.10. Insights on Clinical Applications in Gel-Based Sensors

The gel-based sensors have the ability to transform lab prototypes into clinical applications. These sensors are driven by their features, such as biocompatibility and the ability to be compatible with human tissues [143,144]. These gel-based sensors are based on hydrogels or organogels that enable efficient intimate-to-skin and robust signal transductions. This property of gel-based sensors makes them promising for the healthcare revolution. Among many features, their most clinically impactful feature is their ability to engage in non-invasive health monitoring. These sensors have the ability to monitor biochemical signals like sweat or interstitial fluid. Some sensors are robust enough to engage in non-invasive testing of glucose or heartbeat pulse wave tracking. Another application of these sensors are wound care or tissue regeneration. For example, good hydrogel dressings with integrated sensors can monitor pH, temperature, or moisture. These are regarded as critical parameters that indicate inflections or the healing process. These sensors are especially useful for diabetic ulcers, where early detection of infection can significantly improve patient health and reduce hospital admissions rates. The high stretchability and low modulus also allow them to mimic the mechanical strength of human skin [145]. This makes them ideal for integration into electronic skin systems. These sensors can provide real-time feedback on motion, muscle activity, and pressure distribution. They also assist in providing real-time feedback on patients recovering from injuries or those using prosthetic limbs. Moreover, in clinical trials, these gel-based biosensors are developed to monitor point-of-care testing. They have the ability to attach to biomarkers associated with diseases like cancer. Therefore, these sensors have the ability to reduce the reliance on big labs, thereby enabling faster and more accessible diagnostics with limited cost. In addition to these merits, there are various limitations to the clinical adoption of these sensors. These are long-term stability, filler dispersion, and biofouling resistance. Moreover, ensuring regulatory compliance and data monitoring are critical before scaling of their production [146]. Finally, combinations of these sensors with AI for predictive analytics are expected to further enhance their clinical utility. So, they offer early diagnosis, bridging the gaps between wearables and clinical healthcare systems.

### 4.11. Self-Growing Living Materials or Ultra-Tough Triple-Network Hydrogels

Self-growing living materials and triple-network hydrogels are two critical frontiers in the field of gel-based sensors. These living cells, like bacteria, fungi, etc., exhibit growth, self-repair, and environmental responsiveness. This behavior enables a new class of adaptive sensors for these natural or engineered cells. In terms of sensing, these living components can respond to chemical, mechanical, or biological stimuli with electrical outputs. These outputs are electrical conductivity, fluorescence, or metabolic activity. For example, engineered microbial networks embedded in hydrogels can detect toxins, pH changes, or biomolecules [147]. One of the key merits of these systems is their ability to self-regenerate. Unlike traditional gels, these systems have the ability to repair micro-cracks, restore conductivity, and even improve the sensitivity of the system. Thus, these sensors containing engineered cells are useful for various biomedical applications. However, there are some challenges, such as cell viability, controlled growth, and preventing contamination. Therefore, balancing biological activity with electronic functionality is critical to explore these multifunctionalities.

Similarly, the triple-networking system is an advanced version of the double-network system. These triple-networking systems are designed to achieve high mechanical strength, toughness, and fatigue resistance. The three network systems include a rigid, brittle network, a soft, stretchable network, and a reversible network. Here, the brittle network fractures under stress to dissipate energy [148]. The soft network maintains structural integrity. The third network, or reversible network, contains self-healing agents that enable self-recovery. These aspects of the three-network system achieve synergism to provide multi-functional features. These features include high mechanical strength, high stretchability, good durability, and finally, a robust self-healing ability. Here, an ultra-tough structure ensures stable electrical pathways even under large strains. This aspect helps to reduce hysteresis losses and improve signal stability. Finally, compared to traditional hydrogels, the triple network hydrogels have various merits. These are high fracture energy, low hysteresis loss, enhanced fatigue resistance, and self-healing ability. Therefore, a self-growing living system with triple networking presents a great hybrid system [149]. These hybrids combine biological intelligence along with mechanical robustness. These results are the sensors that ensure high stability and also evolve with self-healing mechanisms to perform even at extreme conditions.

## 5. Conclusions, Challenges, and Future Prospects

This work presents recent advancements in material design, fabrication techniques, and functional integration that are critical for sensing applications. These developments have significantly enhanced the performance and durability of gel-based sensors. Innovations such as the incorporation of conductive nanofillers, double-network structures, and self-healing mechanisms have improved mechanical robustness under extreme deformations. The study also highlights the development of novel gel systems with enhanced mechanical strength, electrical conductivity, and environmental resilience. It emphasizes that strategies such as nanomaterial reinforcement, double-network architectures, and self-healing functionalities play a crucial role in improving sensor performance and durability. Moreover, advances in fabrication techniques have enabled the creation of highly customizable and scalable sensor designs, facilitating seamless integration into wearable devices and soft robotic systems.

In addition, this work provides insights into the ability of these sensors to operate under complex mechanical deformations while maintaining sensitivity and reliability, underscoring their importance in next-generation electronics. However, further research is needed to address challenges related to long-term operational stability, large-scale manufacturing, and biocompatibility under real-world conditions. Future directions may include the development of fully self-powered systems, improved environmental tolerance, and advanced multifunctional sensing platforms. Emerging fabrication strategies, including 3D printing, offer precise structural control and scalable production. Furthermore, gel-based sensors now demonstrate multifunctionality, including strain, pressure, temperature, and biochemical sensing, making them highly suitable for applications in health monitoring, human–machine interfaces, and environmental sensing.

### Economic Barriers or Industrial Roadmaps

This review supports the promising pathways and scenarios for the use of gel-based sensors. This study also describes various merits and challenges of these sensors in their path to commercialization. By understanding these facts after conducting a literature survey, this work further reports the economic barriers and their industrial roadmaps in order to commercialize. Some of the economic barriers are material cost, manufacturing complexity, durability, integration cost, optimization cost, and data processing cost. For material cost, most high-performance sensors rely on expensive materials like MXene during their fabrication. Although these sensors are effective for use on a lab scale, the cost factor limits their large-scale use. Additionally, maintaining uniform dispersion and reproducibility at scale introduces further processing expenses. For manufacturing complexity, many sensors based on gels are involved in a lengthy fabrication process. These steps are polymer synthesis, filler functionalization, crosslinking, encapsulation, and electrode integration, etc. Moreover, the use of expensive processing tools like 3D printing or bioprinting further adds cost barriers to their scalability. For durability prospects, the gel-based sensors face challenges like dehydration, swelling, or freezing, thereby limiting their mechanical stability for long-term operations. These challenges limit the use of these sensors at a large scale for real-world applications. For the integration and optimization of the cost of the sensor, they often rely on protective encapsulation layers. These additional components increase system complexity and cost, especially for wearable or biomedical applications. Moreover, unlike traditional sensors, the gel-based sensors lack well-established standards. For example, they do not have standards on performance metrics, reliability testing, and safety certification. This leads to slow bureaucracy and limits the large-scale use of these sensors for different applications.

Similarly, the industrial roadmap prototypes are briefly proposed for the short term, mid-term, and long term. The short-term prospects for the next 1–3 years focus on low-cost formulations. This involves the use of cheap materials, thereby replacing expensive materials and tools for fabrication. The proposal of targeting high-value applications like soft robotics research or other biomedical patches is critical. This follows the development of a hybrid system to ensure the use of these gel-based sensors for real-world applications. Finally, the project should emphasize the proof-of-concept devices for pilot-scale production. The mid-term process for 3–7 years involves the optimization and calibration process. This step is geared towards the optimization of the fabrication process that involves the use of roll-to-roll coating, screen printing, or solution casting. This follows the improvement of the stability of sensors in relation to dehydration, swelling, or self-healing chemistries. Then, well-established protocols and guidelines are followed for optimizing these prototypes for healthcare and wearable technologies. In the next step, edge AI is integrated into these sensors for signal processing and to reduce data calibration costs. Finally, for the long term, from 7–15 years, the focus is on adoption for mass communication and ecosystem integration. These long-term targets aim to achieve cost parity through material substitution and large-scale production. This will develop the prototype into a fully integrated system that is multifunctional in nature. In the next step, these sensors will expand into large-scale markets like electronic skin and smart textiles. Finally, these sensor protocols will achieve supply chain and industrial standards for wide-scale adoption.

## Figures and Tables

**Table 1:** Overview of the present work.

Materials and Processing	Properties	Applications	
Innovative Materials Novel Fillers Innovative Gel Matrix	Properties Mechanical Electro Mechanical Electrical Thermal	Platform Optimization Innovative Material Testing Novel Sensing Integration Additive Manufacturing Environment Monitoring	Wearable Self-Powered Sensors Electromechanical Properties Output Voltage Power Density
Fabrication Molding 3D/4D Printing Bio-Printing Multifunctional Design Mechanisms Triboelectric Piezoelectric Hybrid System	Main Features High Mechanical Strength High Gauge Factors Optimum Stiffness Improved Flexibility Robust Thermal Stability High Electrical Conductivity	Healthcare Heart Beat Monitoring Mental Health Fitness Trackers Tissue Engineering Bio-Compatibility	Human Machine Interfaces Electronic Skin Soft-Robotics Pressure and Tactile Sensing Photo-Actuators

## Data Availability

Not applicable.
